# Supplementary material for: P53-dependent hypusination of eIF5A affects mitochondrial translation and senescence immune surveillance
Source: Nat Commun. 2024 Aug 28;15:7458. doi: 10.1038/s41467-024-51901-w (PMC11358140; doi:10.1038/s41467-024-51901-w)
Supplement: Supplementary file 1 — Supplementary Information [file 41467_2024_51901_MOESM1_ESM.pdf]

Supplementary information

P53-dependent hypusination of eIF5A affects mitochondrial translation and senescence immune surveillance

Xiangli Jiang[1,2#], Ali Hyder Baig[1#], Giuliana Palazzo[1,3], Rossella Del Pizzo[1,3], Toman Bortecen[4,3], Sven Groessl[5,3], Esther A. Zaal[6], Cinthia Claudia Amaya Ramirez[1], Alexander Kowar[1,3], Daniela Aviles-Huerta[1,3], Celia R. Berkers[6], Wilhelm Palm[5], Darjus Tschaharganeh[7,8], Jeroen Krijgsveld[4,9], Fabricio Loayza-Puch[1,*]

[#]Equal contribution, *Correspondence to f.loayza-puch@dkfz-heidelberg.de

1 Translational Control and Metabolism, German Cancer Research Center (DKFZ), Heidelberg, Germany, 69120 Heidelberg, Germany.
2 Department of Thoracic Oncology, Tianjin Medical University Cancer Institute & Hospital, National Clinical Research Center for Cancer, Key Laboratory of Cancer Prevention and Therapy, Tianjin, Tianjin's Clinical Research Center for Cancer, Tianjin 300060, China
3 Faculty of Biosciences, University of Heidelberg, Heidelberg, Germany
4 Proteomics of Stem Cells and Cancer, German Cancer Research Center (DKFZ), Im Neuenheimer Feld 581, Heidelberg, Germany.
5 Division of Cell Signaling and Metabolism, German Cancer Research Center (DKFZ), Im Neuenheimer Feld 280, Heidelberg, Germany
6 Division of Cell Biology, Metabolism and Cancer, Department Biomolecular Health Sciences, Faculty of Veterinary Medicine, Utrecht University, 3584 CL Utrecht, The Netherlands.
7 Cell Plasticity and Epigenetic Remodeling, German Cancer Research Center (DKFZ), Im Neuenheimer Feld 280, Heidelberg, Germany
8 Institute of Pathology, University Hospital Heidelberg, Im Neuenheimer Feld 224, Heidelberg, Germany
9 Medical Faculty, University of Heidelberg, Heidelberg, Germany

Supplementary Figures 1 to 6:

Supplementary figure 1

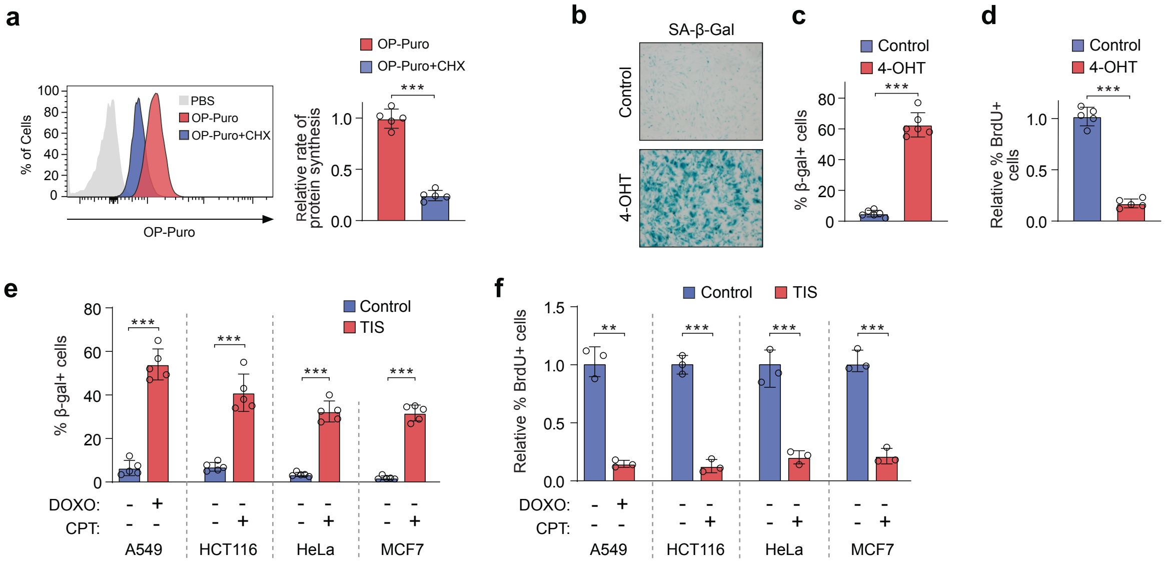

Supplementary Figure 1. Therapy induced senescent (TIS) cells show elevated rates of protein synthesis.

a, OP-Puro incorporation in BJ human fibroblasts. Incorporation in BJ cells in culture was blocked by cycloheximide (CHX, 100 µg/ml) treatment for 1 hour. Data represent mean ± SD from biologically independent experiments (*n* = 5). *p*-values were calculated using a two-tailed unpaired *t*-test. ***$P < 0.001$.

b, Senescence associated beta-galactosidase (SA-β-gal) staining in control and 4-OHT-treated BJ/ET/Ras^{V12}ER cells (BJ-Ras-ER). Cells were treated with 4-OHT for 12 days.

c, Quantification of SA-β-gal expression in control and senescent BJ-Ras-ER cells. Data represent mean ± SD from biologically independent experiments (*n* = 5). *p*-values were calculated using a two-tailed unpaired *t*-test. ***$P < 0.001$.

d, BrdU incorporation assay in proliferating and senescent BJ-Ras-ER cells. Data represent mean ± SD from biologically independent experiments (*n* = 6). *p*-values were calculated using a two-tailed unpaired *t*-test. (*n* = 6); ***$P < 0.001$.

e, Quantification of SA-β-gal expression in proliferating and senescent A549, HCT116, HeLa, and MCF7 cells treated either with doxorubicin (DOXO) or camptothecin (CPT) for 7 days. Data represent mean ± SD from biologically independent experiments (*n* = 5). *p*-values were calculated using a two-tailed unpaired *t*-test. ***$P < 0.001$.

f, Quantification of BrdU incorporation in proliferating and senescent A549, HCT116, HeLa, and MCF7 cells treated either with doxorubicin (DOXO) or camptothecin (CPT). Data represent mean ± SD from biologically independent experiments (*n* = 3). *p*-values were calculated using a two-tailed unpaired *t*-test. **$P < 0.001$; ***$P < 0.001$.

Source data including exact *p*-values are provided as Source Data file.

Supplementary figure 2

a

Genes included in the CRISPR-Cas9 screen		
ANKHD1-EIF4EBP3	*EIF2B4*	*EIF4E*
EEF1A1	*EIF2B5*	*EIF4E1B*
EEF1A2	*EIF2D*	*EIF4E2*
EEF1B2	*EIF2S1*	*EIF4E3*
EEF1D	*EIF2S2*	*EIF4EBP1*
EEF1E1	*EIF2S3*	*EIF4EBP2*
EEF1G	*EIF3A*	*EIF4EBP3*
EEF2	*EIF3B*	*EIF4ENIF1*
EEF2K	*EIF3C*	*EIF4G1*
EEF2KMT	*EIF3CL*	*EIF4G2*
EEFSEC	*EIF3D*	*EIF4G3*
EIF1A	*EIF3E*	*EIF4H*
EIF1AB	*EIF3F*	*EIF5*
EIF1AD	*EIF3G*	*EIF5A*
EIF1AX	*EIF3H*	*EIF5A2*
EIF1AY	*EIF3I*	*EIF5AL1*
EIF2A	*EIF3J*	*EIF5B*
EIF2AK1	*EIF3K*	*EIF6*
EIF2AK2	*EIF3L*	*ETF1*
EIF2AK3	*EIF3M*	*ETFA*
EIF2AK4	*EIF4A1*	*ETFB*
EIF2B1	*EIF4A2*	*ETFDH*
EIF2B2	*EIF4A3*	
EIF2B3	*EIF4B*	

b

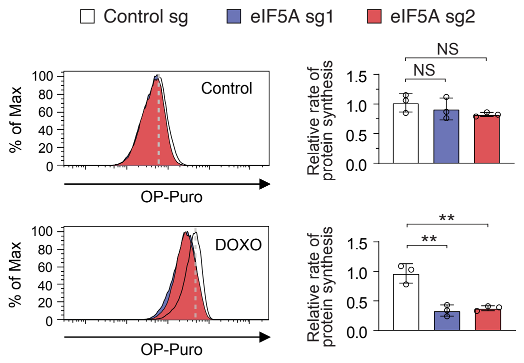

c

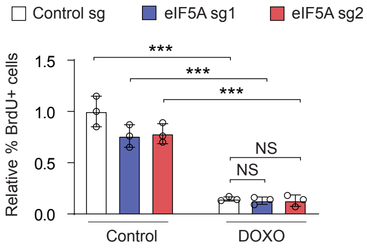

d

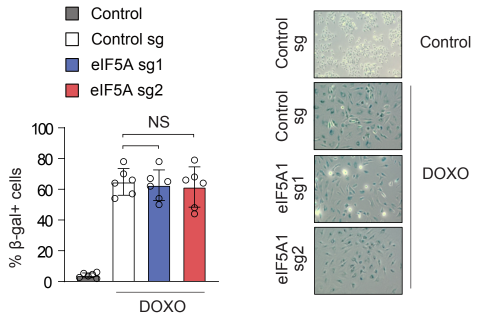

Supplementary Figure 2. eIF5A is required to sustain elevated rates of protein synthesis in TIS cells.

a, The 70 human translation factors targeted in our CRISPR-Cas9 screen.

b, Quantification of OP-Puro in proliferating and senescent A549 cells expressing either a control sgRNA or sgRNAs targeting eIF5A. Data represent mean ± SD from biologically independent experiments (*n* = 3). *p*-values were calculated using a two-tailed unpaired *t*-test. NS, not significant; $^{**}P < 0.01$.

c, BrdU incorporation in A549 cells after 7 days of doxorubicin treatment. Cells were transduced with virus containing sgRNAs targeting eIF5A. Data represent mean ± SD from biologically independent experiments (*n* = 3). *p*-values were calculated using a two-tailed unpaired *t*-test. NS, not significant; $^{***}P < 0.001$.

d, Senescence associated β-galactosidase assay in A549 treated with doxorubicin for 7 days. Cells were transduced with virus containing sgRNAs targeting eIF5A. Data represent mean ± SD from biologically independent experiments (*n* = 6). *p*-values were calculated using a two-tailed unpaired *t*-test. NS, not significant.

Source data including exact *p*-values are provided as Source Data file.

Supplementary Figure 3

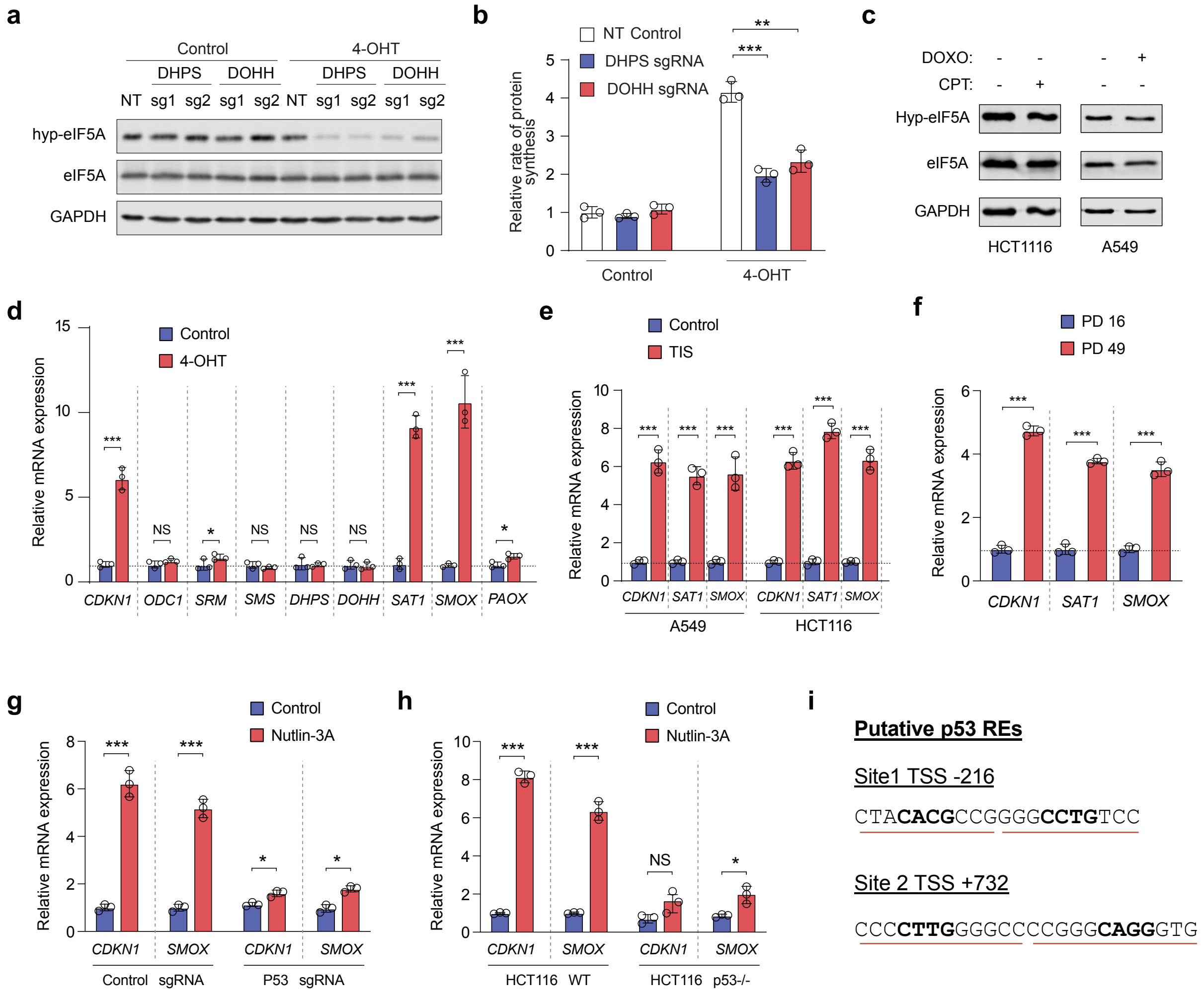

Supplementary Figure 3. p53 regulates the expression of components of the polyamine pathway in TIS cells.

a, Immunoblotting assay from protein extracts of proliferating and OIS BJ-Ras-ER cells transduced with sgRNA targeting DHPS or DOHH. Representative of 3 independent experiments.

b, OP-Puro incorporation assay in proliferating and OIS BJ-Ras-ER cells transduced with sgRNA targeting DHPS or DOHH. Data represent mean ± SD from biologically independent experiments (n = 3). p-values were calculated using a two-tailed unpaired t-test. NS, not significant; **$P < 0.01$; ***$P < 0.001$.

c, Immunoblotting of hypusinated eIF5A and total eIF5A in proliferating and senescent A549 and HCT116 cells treated either with either doxorubicin (DOXO) or camptothecin (CPT). One representative of $n = 3$ biologically independent experiments.

d, qRT-PCR analysis of all the genes involved in the polyamine pathway in proliferating (control) and OIS IMR90-Ras-ER cells (4-OHT). Data represent mean ± SD from biologically independent experiments (n = 3). p-values were calculated using a two-tailed unpaired t-test. NS, not significant; **$P < 0.05$; ***$P < 0.001$.

e-f, qRT-PCR of CDKN1A, SAT1, and SMOX mRNAs in proliferating and senescent A549 and HCT116 cells treated either with doxorubicin (DOXO) or camptothecin (CPT), respectively (e) and in IMR-90 cells undergoing replicative senescence. PD, population doubling. Data represent mean ± SD from biologically independent experiments (n = 3). p-values were calculated using a two-tailed unpaired t-test. ***$P < 0.001$.

g-h, qRT-PCR analysis of SMOX expression upon 24 hrs of Nutlin 3A treatment in BJ cells transduced with either a control sgRNA or an sgRNA targeting p53 (d) and HCT116 WT ($p53^{+/+}$) and $p53^{-/-}$ cells (e). Data represent mean ± SD from biologically independent experiments (n = 3). p-values were calculated using a two-tailed unpaired t-test. *$P < 0.05$; ***$P < 0.001$.

i, Putative p53 response elements (REs) at -216 and +732 bp upstream and downstream of the SMOX gene transcriptional start site (TSS).

Source data including exact p-values are provided as Source Data file.

Supplementary Figure 4

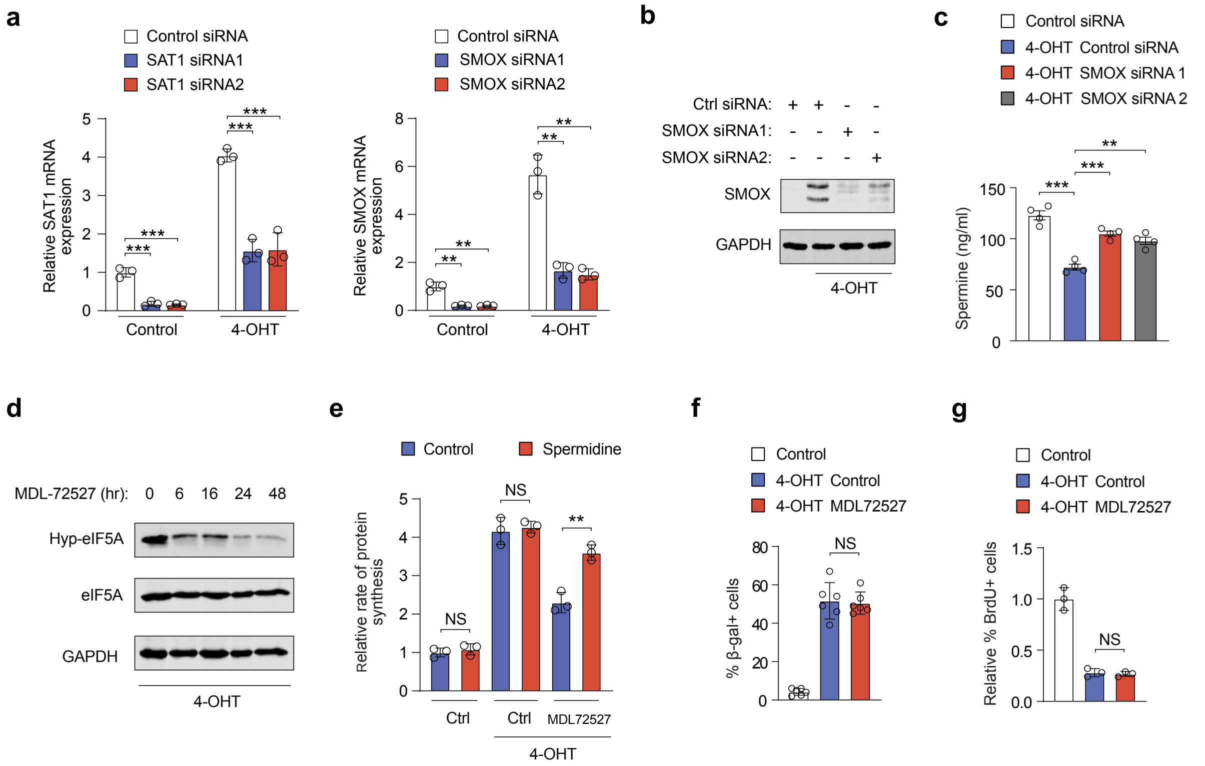

Supplementary Figure 4. SMOX modulates eIF5A hypusination and protein synthesis in OIS.

a, mRNA expression of SAT1 and SMOX measured by qPCR in BJ-Ras-ER cells transfected with the indicated siRNAs. Data represent mean ± SD from biologically independent experiments (n = 3). p-values were calculated using a two-tailed unpaired t-test. ** $P < 0.01$; *** $P < 0.001$.

b, Immunoblotting assay from cell extracts of proliferating and senescent (4-OHT) BJ-Ras-ER cells transfected either with Control siRNA or with siRNAs targeting SMOX. One representative of $n = 3$ biologically independent experiments.

c, Total spermine levels measured in proliferating and senescent (4-OHT) BJ-Ras-ER cells transfected either with Control siRNA or with siRNAs targeting SMOX. Data represent mean ± SD from biologically independent experiments (n = 3). p-values were calculated using a two-tailed unpaired t-test. ** $P < 0.01$; *** $P < 0.001$;

d, Western blot analysis of lysates from OIS BJ-Ras-ER cells treated with the SMOX inhibitor MDL-52727 (50 µM) probed for hyp-eIF5A, eIF5A, and GAPDH expression. One representative of $n = 3$ biologically independent experiments.

e, OP-Puro incorporation in OIS BJ-Ras-ER cells treated with the SMOX inhibitor MDL-52727 (50 µM) and/or spermidine (10 µM). Data represent mean ± SD from biologically independent experiments (n = 3). p-values were calculated using a two-tailed unpaired t-test. NS, not significant; ** $P < 0.01$.

f, Quantification of SA-β-gal positive cells in proliferating and senescent BJ-Ras-ER cells treated with MDL-52727 (50 µM). Data represent mean ± SD from biologically independent experiments (n = 6). p-values were calculated using a two-tailed unpaired t-test. NS, not significant.

g, BrdU incorporation in BJ-Ras-ER cells treated with the SMOX inhibitor MDL-52727 (50 µM). Data represent mean ± SD from biologically independent experiments (n = 3). p-values were calculated using a two-tailed unpaired t-test. NS, not significant.

Source data including exact p-values are provided as Source Data file.

Supplementary Figure 5

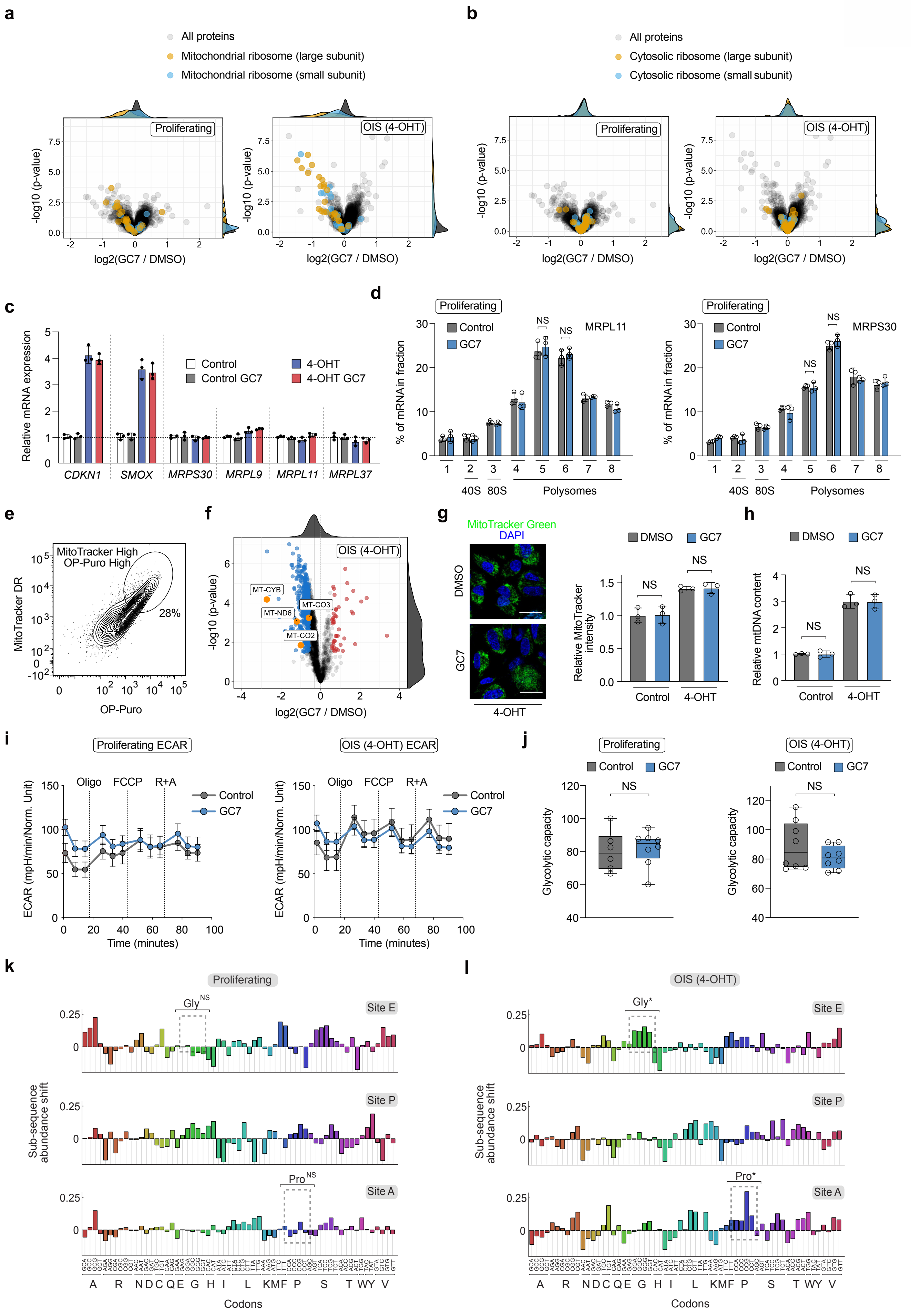

Supplementary Figure 5. eIF5A promotes the translation of mitochondrial ribosomal proteins and mitochondrially-encoded genes in senescent cells.

a-b, Volcano plots depict differentially expressed proteins in proliferating and OIS BJ-Ras-ER cells treated with GC7 (10 μM) for 12 hours. Mitochondrial ribosomal proteins (a) and cytosolic ribosomal proteins (b) are highlighted.

c, qRT-PCR of selected mitochondrial ribosomal proteins in proliferating and OIS BJ-Ras-ER cells treated with treated with GC7 (10 μM) for 24 hours. Data represent mean ± SD from biologically independent experiments (n = 3). p-values were calculated using a two-tailed unpaired t-test.

d, Polysome analyses of selected mitochondrial ribosomal proteins (MRPL11 and MRPS30) in proliferating BJ-Ras-ER cells treated with GC7 (10 μM) for 12 hours. Data represent mean ± SD from biologically independent experiments (n = 3). p-values were calculated using a two-tailed unpaired t-test. NS, not significant.

e, Bivariate flow cytometry density plot of OIS BJ-Ras-ER cells. Cells were stained with MitoTracker DR and Alexa Fluor 488-Azide.

f, OIS BJ-Ras-ER cells were treated with GC7 (10 μM) for 12 hours and subjected to both AHA and pSILAC labelling for additional 4 hours. The volcano plot shows $\log_2$-transformed fold differences of newly synthesized proteins between treated (GC7) and control cells (DMSO). Proteins with a log2 fold difference > 0.5 and adj. p-value < 0.05, representing proteins with a higher synthesis rate in GC7-treated cells, are highlighted in red. Proteins with a log2 fold difference < −0.5 and adj. p-value < 0.05, representing proteins with a lower synthesis rate in GC7-treated cells, are highlighted in blue. Proteins encoded by mtDNA are highlighted in orange.

g, Representative micrographs of MitoTracker Green-labeled OIS BJ-Ras-ER cells treated either with DMSO or GC7 (10 μM) for 12 hours. Blue shows nuclei staining with DAPI. The scale bar is 25 μm. One representative of n = 3 biologically independent experiments. The right panel shows the relative fluorescence intensities of MitoTracker Green, quantified in proliferating and OIS BJ-Ras-ER cells treated with DMSO or GC7 (10 μM) for 12 hours. Values are expressed relative to the fluorescence intensities of MitoTracker Green in control proliferating BJ-Ras-ER cells. The data represents mean ± SD from biologically independent experiments (n = 3). p-values were calculated using a two-tailed unpaired t-test. NS, not significant.

h, mtDNA copy number analysis by qPCR in proliferating and OIS BJ-Ras-ER cells. Cells were treated with or without GC7 (10 μM) for 12 hours. Data represent mean ± SD from biologically independent experiments (n = 3). p-values were calculated using a two-tailed unpaired t-test. NS, not significant.

i-j, Extracellular acidification rates (ECAR) measurements were assessed by Seahorse Extracellular Flux Analyzer (EFA) in proliferating and OIS BJ-Ras-ER cells treated with GC7 (10 μM) for 16 hours. Measurements were made under basal conditions or following the addition of oligomycin (Oligo, 0.5 μM), the uncoupler FCCP (1 μM), or the electron transport inhibitors Rotenone and Antimycin A (R+A, 0.5 μM each).). (j) Data are presented as box plots (*n* = 8), center line represents the median, upper bound represents the 75th percentile and lower bound represents the 25th percentile. Whiskers represent minimum and maximum values. *p*-values were calculated using a two-tailed unpaired *t*-test. NS, not significant; ***$P < 0.001$.

k-l, Cytosolic subsequence analysis examined the codons at 9, 12 and 15 nucleotides from the 5′-ends of RPFs in proliferating (j) and OIS (k) BJ-Ras-ER cells treated with GC7 (10 μM) for 18 hours. Data represents RPF codon occupancy frequencies between samples. Frequencies are normalized in a gene-level manner. *Out-of-frame analysis $P < 0.001$ for the CCT, CCG Pro codons and for the GGA, GGC, GGG, GGT Gly codons. NS, not significant. Source data including exact *p*-values are provided as Source Data file.

Supplementary Figure 6

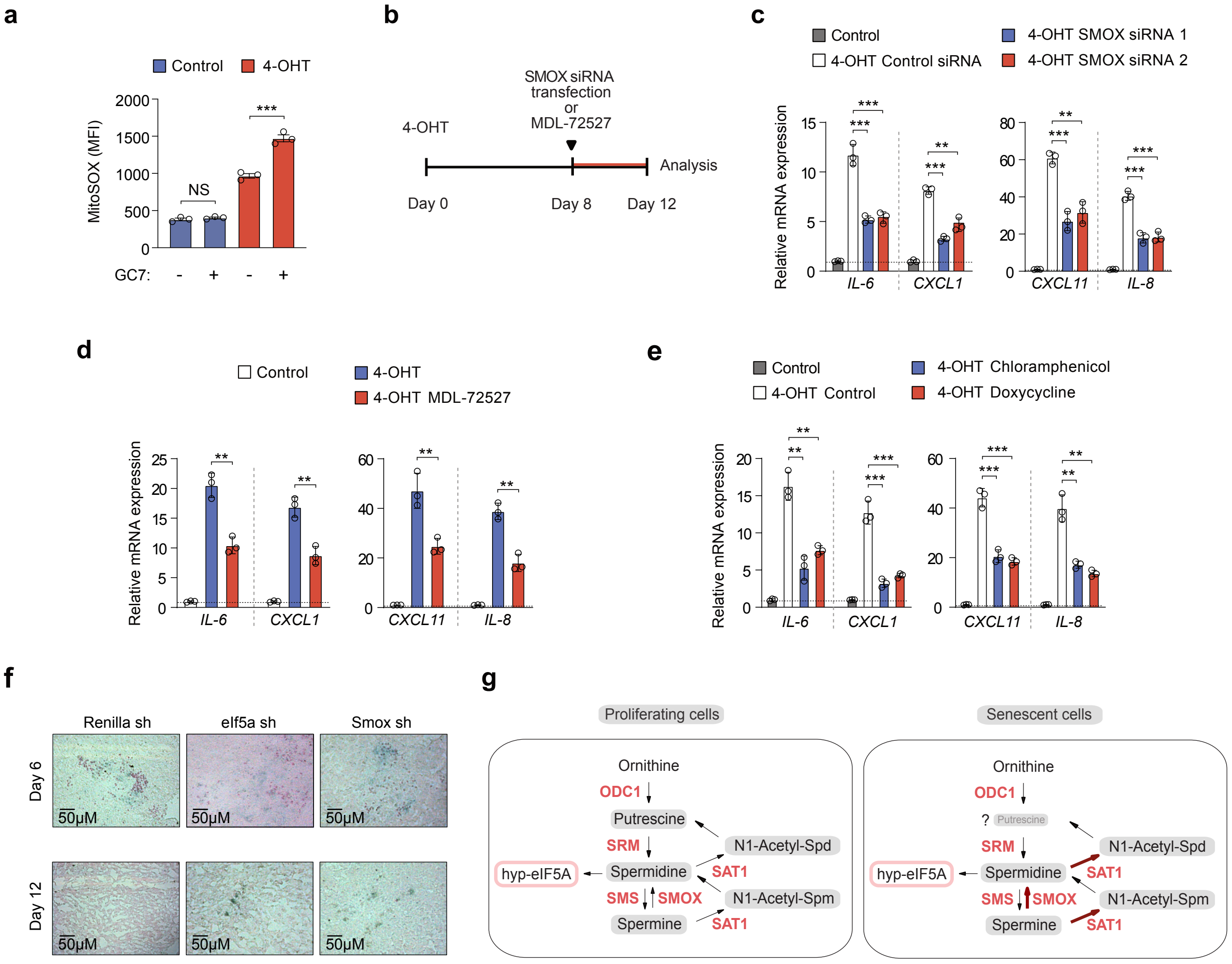

Supplementary Figure 6. SMOX inhibition suppresses the SASP in already established senescent cells.

a, MitoSOX flow cytometry measurements (MFI, median fluorescence intensity) in proliferating and senescent (4-OHT) BJ-Ras-ER cells treated with either control or GC7 (10 μM) for 12 hours. Data represent mean ± SD from biologically independent experiments (*n* = 3). *p*-values were calculated using a two-tailed unpaired *t*-test. NS, not significant; ****P* < 0.01.

b, Scheme of the experimental design for the siRNA transfection and drug treatments in already established BJ-Ras-ER senescent cells.

c, Quantitative real-time polymerase chain reaction (qRT-PCR) of selected pro-inflammatory cytokines in OIS BJ-Ras-ER cells transfected with either non-targeting (Control) or SMOX siRNAs. Data represent mean ± SD from biologically independent experiments (*n* = 3). *p*-values were calculated using a two-tailed unpaired *t*-test. ***P* < 0.01; ****P* < 0.001.

d, Expression of the indicated SASP genes in proliferating and OIS BJ-Ras-ER cells treated with MDL-72527 (50 μM) as described in (a). Data represent mean ± SD from biologically independent experiments (*n* = 3). *p*-values were calculated using a two-tailed unpaired t-test. ***P* < 0.01; ****P* < 0.001.

e, Expression of pro-inflammatory genes in OIS BJ-Ras-ER cells treated with Chloramphenicol (100 μM) or Doxycycline (5 μM) as described in (a). Data represent mean ± SD from biologically independent experiments (*n* = 3). *p*-values were calculated using a two-tailed unpaired *t*-test. ***P* < 0.01; ****P* < 0.001.

f, SA-β-gal staining on frozen tissue sections of livers harvested at days 6 and 12 after transduction with the indicated vectors. One representative of *n* = 3 biologically independent experiments.

g, Schematic depicting the rewiring of the polyamine pathway in senescent cells. In proliferating cells, the abundance of putrescine is sufficient to synthesize spermidine and sustain eIF5A hypusination levels. In senescent cells, levels of putrescine significantly decrease via an unknown mechanism. The senescent program activates the expression of SMOX and SAT1 to recycle polyamines, sustaining spermidine and eIF5A hypusination levels. Source data including exact *p*-values are provided as Source Data file.
