# Supplementary material for: P53-dependent hypusination of eIF5A affects mitochondrial translation and senescence immune surveillance
Source: Nat Commun. 2024 Aug 28;15:7458. doi: 10.1038/s41467-024-51901-w (PMC11358140; doi:10.1038/s41467-024-51901-w)
Supplement: Supplementary file 3 — Description of Additional Supplementary Files [file 41467_2024_51901_MOESM3_ESM.pdf]

Description of Additional Supplementary Files

Supplementary Data 1: Results of the OP-Puro based screen in OIS BJ-Ras-ER

Supplementary Data 2: Results of the whole proteome analysis in proliferating BJ-Ras-ER

Supplementary Data 3: Results of the whole proteome analysis in OIS BJ-Ras-ER

Supplementary Data 4: eIF5A tripeptide motifs present in mitochondrial and cytosolic ribosomal proteins

Supplementary Data 5: Oligonucleotides used in this study
