# Supplementary material for: P53-dependent hypusination of eIF5A affects mitochondrial translation and senescence immune surveillance
Source: Nat Commun. 2024 Aug 28;15:7458. doi: 10.1038/s41467-024-51901-w (PMC11358140; doi:10.1038/s41467-024-51901-w)
Supplement: Supplementary file 7 — Supplementary Data 4 [file 41467_2024_51901_MOESM7_ESM.rtf]

Supplementary Dataset 4. eIF5A tripeptide motifs present in mitochondrial and cytosolic ribosomal proteins.Mitochondrial Ribosome>MRPS2MATSSAALPRILGAGARAPSRWLGFLGKATPRPARPSRRTLGSATALMIRESEDSTDFNDKILNEPLKHSDFFNVKELFSVRSLFDARVHLGHKAGCRHRFMEPYIFGSRLDHDIIDLEQTATHLQLALNFTAHMAYRKGIILFISRNRQFSYLIENMARDCGEYAHTRYFRGGMLTNARLLFGPTVRLPDLIIFLHTLNNIFEPHVAVRDAAKMNIPTVGIVDTNCNPCLITYPVPGNDDSPLAVHLYCRLFQTAITRAKEKRQQVEALYRLQGQKEPGDQGPAHPPGADMSHSL >MRPS5MATAVRAVGCLPVLCSGTAGHLLGRQCSLNTLPAASILAWKSVLGNGHLSSLGTRDTHPYASLSRALQTQCCISSPSHLMSQQYRPYSFFTKLTADELWKGALAETGAGAKKGRGKRTKKKKRKDLNRGQIIGEGRYGFLWPGLNVPLMKNGAVQTIAQRSKEEQEKVEADMIQQREEWDRKKKMKVKRERGWSGNSWGGISLGPPDPGPCGETYEDFDTRILEVRNVFTMTAKEGRKKSIRVLVAVGNGKGAAGFSIGKATDRMDAFRKAKNRAVHHLHYIERYEDHTIFHDISLRFKRTHIKMKKQPKGYGLRCHRAIITICRLIGIKDMYAKVSGSINMLSLTQGLFRGLSRQETHQQLADKKGLHVVEIREECGPLPIVVASPRGPLRKDPEPEDEVPDVKLDWEDVKTAQGMKRSVWSNLKRAAT>MRPS6MPRYELALILKAMQRPETAATLKRTIEALMDRGAIVRDLENLGERALPYRISAHSQQHNRGGYFLVDFYAPTAAVESMVEHLSRDIDVIRGNIVKHPLTQELKECEGIVPVPLAEKLYSTKKRKK>MRPS7MAAPAVKVARGWSGLALGVRRAVLQLPGLTQVRWSRYSPEFKDPLIDKEYYRKPVEELTEEEKYVRELKKTQLIKAAPAGKTSSVFEDPVISKFTNMMMIGGNKVLARSLMIQTLEAVKRKQFEKYHAASAEEQATIERNPYTIFHQALKNCEPMIGLVPILKGGRFYQVPVPLPDRRRRFLAMKWMITECRDKKHQRTLMPEKLSHKLLEAFHNQGPVIKRKHDLHKMAEANRALAHYRWW>MRPS9MAAPCVSYGGAVSYRLLLWGRGSLARKQGLWKTAAPELQTNVRSQILRLRHTAFVIPKKNVPTSKRETYTEDFIKKQIEEFNIGKRHLANMMGEDPETFTQEDIDRAIAYLFPSGLFEKRARPVMKHPEQIFPRQRAIQWGEDGRPFHYLFYTGKQSYYSLMHDVYGMLLNLEKHQSHLQAKSLLPEKTVTRDVIGSRWLIKEELEEMLVEKLSDLDYMQFIRLLEKLLTSQCGAAEEEFVQRFRRSVTLESKKQLIEPVQYDEQGMAFSKSEGKRKTAKAEAIVYKHGSGRIKVNGIDYQLYFPITQDREQLMFPFHFVDRLGKHDVTCTVSGGGRSAQAGAIRLAMAKALCSFVTEDEVEWMRQAGLLTTDPRVRERKKPGQEGARRKFTWKKR>MRPS10MAARTAFGAVCRRLWQGLGNFSVNTSKGNTAKNGGLLLSTNMKWVQFSNLHVDVPKDLTKPVVTISDEPDILYKRLSVLVKGHDKAVLDSYEYFAVLAAKELGISIKVHEPPRKIERFTLLQSVHIYKKHRVQYEMRTLYRCLELEHLTGSTADVYLEYIQRNLPEGVAMEVTKTQLEQLPEHIKEPIWETLSEEKEESKS>MRPS11-1MQAVRNAGSRFLRSWTWPQTAGRVVARTPAGTICTGARQLQDAAAKQKVEQNAAPSHTKFSIYPPIPGEESSLRWAGKKFEEIPIAHIKASHNNTQIQVVSASNEPLAFASCGTEGFRNAKKGTGIAAQTAGIAAAARAKQKGVIHIRVVVKGLGPGRLSAMHGLIMGGLEVISITDNTPIPHNGCRPRKARKL>MRPS12-1MSWSGLLHGLNTSLTCGPALVPRLWATCSMATLNQMHRLGPPKRPPRKLGPTEGRPQLKGVVLCTFTRKPKKPNSANRKCCRVRLSTGREAVCFIPGEGHTLQEHQIVLVEGGRTQDLPGVKLTVVRGKYDCGHVQKK>MRPS14MAAFMLGSLLRTFKQMVPSSASGQVRSHYVDWRMWRDVKRRKMAYEYADERLRINSLRKNTILPKILQDVADEEIAALPRDSCPVRIRNRCVMTSRPRGVKRRWRLSRIVFRHLADHGQLSGIQRATW>MRPS15MLRVAWRTLSLIRTRAVTQVLVPGLPGGGSAKFPFNQWGLQPRSLLLQAARGYVVRKPAQSRLDDDPPPSTLLKDYQNVPGIEKVDDVVKRLLSLEMANKKEMLKIKQEQFMKKIVANPEDTRSLEARIIALSVKIRSYEEHLEKHRKDKAHKRYLLMSIDQRKKMLKNLRNTNYDVFEKICWGLGIEYTFPPLYYRRAHRRFVTKKALCIRVFQETQKLKKRRRALKAAAAAQKQAKRRNPDSPAKAIPKTLKDSQ>MRPS16MVHLTTLLCKAYRGGHLTIRLALGGCTNRPFYRIVAAHNKCPRDGRFVEQLGSYDPLPNSHGEKLVALNLDRIRHWIGCGAHLSKPMEKLLGLAGFFPLHPMMITNAERLRRKRAREVLLASQKTDAEATDTEATET>MRPS17MSVVRSSVHARWIVGKVIGTKMQKTAKVRVTRLVLDPYLLKYFNKRKTYFAHDALQQCTVGDIVLLRALPVPRAKHVKHELAEIVFKVGKVIDPVTGKPCAGTTYLESPLSSETTQLSKNLEELNISSAQ>MRPS18AMAALKALVSGCGRLLRGLLAGPAATSWSRLPARGFREVVETQEGKTTIIEGRITATPKESPNPPNPSGQCPICRWNLKHKYNYDDVLLLSQFIRPHGGMLPRKITGLCQEEHRKIEECVKMAHRAGLLPNHRPRLPEGVVPKSKPQLNRYLTRWAPGSVKPIYKKGPRWNRVRMPVGSPLLRDNVCYSRTPWKLYH>MRPS18BMAASVLNTVLRRLPMLSLFRGSHRVQVPLQTLCTKAPSEEDSLSSVPISPYKDEPWKYLESEEYQERYGSRPVWADYRRNHKGGVPPQRTRKTCIRRNKVVGNPCPICRDHKLHVDFRNVKLLEQFVCAHTGIIFYAPYTGVCVKQHKRLTQAIQKARDHGLLIYHIPQVEPRDLDFSTSHGAVSATPPAPTLVSGDPWYPWYNWKQPPERELSRLRRLYQGHLQEESGPPPESMPKMPPRTPAEASSTGQTGPQSAL>MRPS18CMAAVVAVCGGLGRKKLTHLVTAAVSLTHPGTHTVLWRRGCSQQVSSNEDLPISMENPYKEPLKKCILCGKHVDYKNVQLLSQFVSPFTGCIYGRHITGLCGKKQKEITKAIKRAQIMGFMPVTYKDPAYLKDPKVCNIRYRE>MRPS21-1MAKHLKFIARTVMVQEGNVESAYRTLNRILTMDGLIEDIKHRRYYEKPCRRRQRESYERCRRIYNMEMARKINFLMRKNRADPWQGC>MRPS22MAPLGTTVLLWSLLRSSPGVERVCFRARIQPWHGGLLQPLPCSFEMGLPRRRFSSEAAESGSPETKKPTFMDEEVQSILTKMTGLNLQKTFKPAIQELKPPTYKLMTQAQLEEATRQAVEAAKVRLKMPPVLEERVPINDVLAEDKILEGTETTKYVFTDISYSIPHRERFIVVREPSGTLRKASWEERDRMIQVYFPKEGRKILTPIIFKEENLRTMYSQDRHVDVLNLCFAQFEPDSTEYIKVHHKTYEDIDKRGKYDLLRSTRYFGGMVWYFVNNKKIDGLLIDQIQRDLIDDATNLVQLYHVLHPDGQSAQGAKDQAAEGINLIKVFAKTEAQKGAYIELTLQTYQEALSRHSAAS>MRPS23MAGSRLETVGSIFSRTRDLVRAGVLKEKPLWFDVYDAFPPLREPVFQRPRVRYGKAKAPIQDIWYHEDRIRAKFYSVYGSGQRAFDLFNPNFKSTCQRFVEKYTELQKLGETDEEKLFVETGKALLAEGVILRRVGEARTQHGGSHVSRKSEHLSVRPQTALEENETQKEVPQDQHLEAPADQSKGLLPP>MRPS24MAASVCSGLLGPRVLSWSRELPCAWRALHTSPVCAKNRAARVRVSKGDKPVTYEEAHAPHYIAHRKGWLSLHTGNLDGEDHAAERTVEDVFLRKFMWGTFPGCLADQLVLKRRGNQLEICAVVLRQLSPHKYYFLVGYSETLLSYFYKCPVRLHLQTVPSKVVYKYL>MRPS25MPMKGRFPIRRTLQYLSQGNVVFKDSVKVMTVNYNTHGELGEGARKFVFFNIPQIQYKNPWVQIMMFKNMTPSPFLRFYLDSGEQVLVDVETKSNKEIMEHIRKILGKNEETLREEEEEKKQLSHPANFGPRKYCLRECICEVEGQVPCPSLVPLPKEMRGKYKAALKADAQD>MRPS26MLRALSRLGAGTPCRPRAPLVLPARGRKTRHDPLAKSKIERVNMPPAVDPAEFFVLMERYQHYRQTVRALRMEFVSEVQRKVHEARAGVLAERKALKDAAEHRELMAWNQAENRRLHELRIARLRQEEREQEQRQALEQARKAEEVQAWAQRKEREVLQLQEEVKNFITRENLEARVEAALDSRKNYNWAITREGLVVRPQRRDS>MRPS27MAASIVRRGMLLARQVVLPQLSPAGKRYLLSSAYVDSHKWEAREKEHYCLADLASLMDKTFERKLPVSSLTISRLIDNISSREEIDHAEYYLYKFRHSPNCWYLRNWTIHTWIRQCLKYDAQDKALYTLVNKVQYGIFPDNFTFNLLMDSFIKKENYKDALSVVFEVMMQEAFEVPSTQLLSLYVLFHCLAKKTDFSWEEERNFGASLLLPGLKQKNSVGFSSQLYGYALLGEGGVAARATGCVPQHASDMETRLP*QSPSSDGESGCLPRRHKAV*RSARCAGCSAEGSDFS*WGFRGAVPK**RQPGVRKTGGAVRHRGNRAVQASSIPGTI*GLTF*ASSSGQN*VRRSFKSDHPACQGKTLHL*SRGHRHL*AESAAVASRPCTVDPERTATEGASEAGVPGSESSKGICL>MRPS28MAALCRTRAVAAESHFLRVFLFFRPFRGVGTESGSESGSSNAKEPKTRAGGFASALERHSELLQKVEPLQKGSPKNVESFASMLRHSPLTQMGPAKDKLVIGRIFHIVENDLYIDFGGKFHCVCRRPEVDGEEIPERNQGPVAAIRS*TYV*VPGSNNRYNCTRG*CSSLGNPGE*RLKIERRTS*KI>MRPS29-1MMLKGITRLISRIHKLDPGRFLHMGTQARQSIAAHLDNQVPVESPRAISRTNENDPAKHGDQHEGQHYNISPQDLETVFPHGLPPRFVMQVKTFSEACLMVRKPALELLHYLKNTSFAYPAIRYLLYGEKGTGKTLSLCHVIHFCAKQDWLILHIPDAHLWVKNCRDLLQSSYNKQRFDQPLEASTWLKNFKTTNERFLNQIKVQEKYVWNKRESTEKGSPLGEVVEQGITRVRNATDAVGIVLKELKRQSSLGMFHLLVAVDGINALWGRTTLKREDKSPIAPEELALVHNLRKMMKNDWHGGAIVSALSQTGSLFKPRKAYLPQELLGKEGFDALDPFIPILVSNYNPKEFESCIQYYLENNWLQHEKAPTEEGKKELLFLSNANPSLLERHCAYL>MRPS30MAAARCWRPLLRGPRLSLHTAANAAATATETTCQDVAATPVARYPPIVASMTADSKAARLRRIERWQATVHAAESVDEKLRILTKMQFMKYMVYPQTFALNADRWYQYFTKTVFLSGLPPPPAEPEPEPEPEPEPALDLAALRAVACDCLLQEHFYLRRRRRVHRYEESEVISLPFLDQLVSTLVGLLSPHNPALAAAALDYRCPVHFYWVRGEEIIPRGHRRGRIDDLRYQIDDKPNNQIRISKQLAEFVPLDYSVPIEIPTIKCKPDKLPLFKRQYENHIFVGSKTADPCCYGHTQFHLLPDKLRRERLLRQNCADQIEVVFRANAIASLFAWTGAQAMYQGFWSEADVTRPFVSQAVITDGKYFSFFCYQLNTLALTTQADQNNPRKNICWGTQSKPLYETIEDNDVKGFNDDVLLQIVHFLLNRPKEEKSQLLEN>MRPS31MFPRVSTFLPLRPLSRHPLSSGSPETSAAAIMLLTVRHGTVRYRSSALLARTKNNIQRYFGTNSVICSKKDKQSVRTEETSKETSESQDSEKENTKKDLLGIIKGMKVELSTVNVRTTKPPKRRPLKSLEATLGRLRRATEYAPKKRIEPLSPELVAAASAVADSLPFDKQTTKSELLSQLQQHEEESRAQRDAKRPKISFSNIISDMKVARSATARVRSRPELRIQFDEGYDNYPGQEKTDDLKKRKNIFTGKRLNIFDMMAVTKEAPETDTSPSLWDVEFAKQLATVNEQPLQNGFEELIQWTKEGKLWEFPINNEAGFDDDGSEFHEHIFLEKHLESFPKQGPIRHFMELVTCGLSKNPYLSVKQKVEHIEWFRNYFNEKKDILKESNIQFN>MRPS33-1MSSLSEYAFRMSRLSARLFGEVTRPTNSKSMKVVKLFSELPLAKKKETYDWYPNHHTYAELMQTLRFLGLYRDEHQDFMDEQKRLKKLRGKEKPKKGEGKRAAKRK>MRPS34MARKKVRPRLIAELARRVRALREQLNRPRDSQLYAVDYETLTRPFSGRRLPVRAWADVRRESRLLQLLGRLPLFGLGRLVTRKSWLWQHDEPCYWRLTRVRPDYTAQNLDHGKAWGILTFKGKTESEAREIEHVMYHDWRLVPKHEEEAFTAFTPAPEDSLASVPYPPLLRAMIIAERQKNGDTSTEEPMLNVQRIRMEPWDYPAKQEDKGRAKGTPV*>MRPS35MAAAALPAWLSLQSRARTLRAFSTAVYSATPVPTPSLPERTPGNERPPRRKALPPRTEKMAVDQDWPSVYPVAAPFKPSAVPLPVRMGYPVKKGVPMAKEGNLELLKIPNFLHLTPVAIKKHCEALKDFCTEWPAALDSDEKCEKHFPIEIDSTDYVSSGPSVRNPRARVVVLRVKLSSLNLDDHAKKKLIKLVGERYCKTTDVLTIKTDRCPLRRQNYDYAVYLLTVLYHESWNTEEWEKSKTEADMEEYIWENSSSERNILETLLQMKAAEKNMEINKEELLGTKEIEEYKKSVVSLKNEEENENSISQYKESVKRLLNVT>MRPS36MMGSKMASASRVVQVVKPHTPLIRFPDRRDNPKPNVSEALRSAGLPSHSSVISQHSKGSKSPDLLMYQGPPDTAEIIKTLPQKYRRKLVSQEEMEFIQRGGPE>MRPL1MVYQTSLCSCSVNIRVPNRHFAAATKSAKKTKKGAKEKTPDEKKDEIEKIKAYPYMEGEPEDDVYLKRLYPRQIYEVEKAVHLLKKFQILDFTSPKQSVYLDLTLDMALGKKKNVEPFTSVLSLPYPFASEINKVAVFTENASEVKIAEENGAAFAGGTSLIQKIWDDEIVADFYVAVPEIMPELNRLRKKLNKKYPKLSRNSIGRDIPKMLELFKNGHEIKVDEERENFLQTKIATLDMSSDQIAANLQAVINEVCRHRPLNLGPFVVRAFLRSSTSEGLLLKIDPLLPKEVKNEESEKEDA>MRPL2MALCALTRALRSLNLAPPTVAAPAPSLFPAAQMMNNGLLQQPSALMLLPCRPVLTSVALNANFVSWKSRTKYTITPVKMRKSGGRDHTGRIRVHGIGGGHKQRYRMIDFLRFRPEETKSGPFEEKVIQVRYDPCRSADIALVAGGSRKRWIIATENMQAGDTILNSNHIGRMAVAAREGDAHPLGALPVGTLINNVESEPGRGAQYIRAAGTCGVLLRKVNGTAIIQLPSKRQMQVLETCVATVGRVSNVDHNKRVIGKAGRNRWLGKRPNSGRWHRKGGWAGRKIRPLPPMKSYVKLPSASAQS>MRPL3MPGWRLLTQVGAQVLGRLGDGLGAALGPGNRTHIWLFVRGLHGKSGTWWDEHLSEENVPFIKQLVSDEDKAQLASKLCPLKDEPWPIHPWEPGSFRVGLIALKLGMMPLWTKDGQKHVVTLLQVQDCHVLKYTSKENCNGKMATLSVGGKTVSRFRKATSILEFYRELGLPPKQTVKIFNITDNAAIKPGTPLYAAHFRPGQYVDVTAKTIGKGFQGVMKRWGFKGQPATHGQTKTHRRPGAVATGDIGRVWPGTKMPGKMGNIYRTEYGLKVWRINTKHNIIYVNGSVPGHKNCLVKVKDSKLPAYKDLGKNLPFPTYFPDGDEEELPEDLYDENVCQPGAPSITFA>MRPL4-1MLQFVRAGARAWLRPTGSQGLSSLAEEAARATENPEQVASEGLPEPVLRKVELPVPTHRRPVQAWVESLRGFEQERVGLADLHPDVFATAPRLDILHQVAMWQKNFKRISYAKTKTRAEVRGGGRKPWPQKGTGRARHGSIRSPLWRGGGVAHGPRGPTSYYYMLPMKVRALGLKVALTVKLAQDDLHIMDSLELPTGDPQYLTELAHYRRWGDSVLLVDLTHEEMPQSIVEATSRLKTFNLIPAVGEQRAQAPRVRMCRLRC>MRPL4-2MLQFVRAGARAWLRPTGSQGLSSLAEEAARATENPEQVASEGLPEPVLRKVELPVPTHRRPVQAWVESLRGFEQERVGLADLHPDVFATAPRLDILHQVAMWQKNFKRISYAKTKTRAEVRGGGRKPWPQKGTGRARHGSIRSPLWRGGGVAHGPRGPTSYYYMLPMKVRALGLKVALTVKLAQDDLHIMDSLELPTGDPQYLTELAHYRRWGDSVLLVDLTHEEMPQSIVEATSRLKTFNLIPAVGLNVHSMLKHQTLVLTLPTVAFLEDKLLWQDSRYRPLYPFSLPYSDFPRPLPHATQGPAATPYHC>MRPL4-3MLQFVRAGARAWLRPTGSQGLSSLAEEAARATENPEQVASEGLPEPVLRKVELPVPTHRRPVQAWVESLRGFEQERVGLADLHPDVFATAPRLDILHQVAMWQKNFKRISYAKTKTRAEVRGGGRKPWPQKGTGRARHGSIRSPLWRGGGVAHGPRGPTSYYYMLPMKVRALGLKVALTVKLAQDDLHIMDSLELPTGDPQYLTELAHYRRWGDSVLLVDLTHEEMPQSIVEATSRLKTFNLIPAVGLNVHSMLKHQTLVLTLPTVAFLEDKLLWQDSRYRPLYPFSLPYSDFPRPLPHATQGPAATPYHC>MRPL9MAAPVVTAPGRALLRAGAGRLLRGGVQELLRPRHEGNAPDLACNFSLSQNRGTVIVERWWKVPLAGEGRKPRLHRRHRVYKLVEDTKHRPKENLELILTQSVENVGVRGDLVSVKKSLGRNRLLPQGLAVYASPENKKLFEEEKLLRQEGKLEKIQTKAGEATVKFLKSCRLEVGMKNNVKWELNPEIVARHFFKNLGVVVAPHTLKLPEEPITRWGEYWCEVTVNGLDTVRVPMSVVNFEKPKTKRYKYWLAQQAAKAMAPTSPQI>MRPL10-2MISAHCNLHLPGSSDSPASASQVAGITGRLPTLQTVRYGSKAVTRHRRVMHFQRQKLMAVTEYIPPKPAIHPSCLPSPPSPPQEEIGLIRLLRREIAAVFQDNRMIAVCQNVALSAEDKLLMRHQLRKHKILMKVFPNQVLKPFLEDSKYQNLLPLFVGHNMLLVSEEPKVKEMVRILRTVPFLPLLGGCIDDTILSRQGFINYSKLPSLPLVQGELVGGLTCLTAQTHSLLQHQPLQLTTLLDQYIREQREKDSVMSANGKPDPDTVPDS>MRPL11-1MSKLGRAARGLRKPEVGGVIRAIVRAGLAMPGPPLGPVLGQRGVSINQFCKEFNERTKDIKEGIPLPTKILVKPDRTFEIKIGQPTVSYFLKAAAGIEKGARQTGKEVAGLVTLKHVYEIARIKAQDEAFALQDVPLSSVVRSIIGSARSLGIRVVKDLSSEELAAFQKERAIFLAAQKEADLAAQEEAAKK>MRPL12MLPAAARPLWGPCLGLRAAAFRLARRQVPCVCAVRHMRSSGHQRCEALAGAPLDNAPKEYPPKIQQLVQDIASLTLLEISDLNELLKKTLKIQDVGLVPMGGVMSGAVPAAAAQEAVEEDIPIAKERTHFTVRLTEAKPVDKVKLIKEIKNYIQGINLVQAKKLVESLPQEIKANVAKAEAEKIKAALEAVGGTVVLE>MRPL13MSSFSRAPQQWATFARIWYLLDGKMQPPGKLAAMASIRLQGLHKPVYHALSDCGDHVVIMNTRHIAFSGNKWEQKVYSSHTGYPGGFRQVTAAQLHLRDPVAIVKLAIYGMLPKNLHRRTMMERLHLFPDEYIPEDILKNLVEELPQPRKIPKRLDEYTQEEIDAFPRLWTPPEDYRL>MRPL14MAFFTGLWGPFTCVSRVLSHHCFSTTGSLSAIQKMTRVRVVDNSALGNSPYHRAPRCIHVYKKNGVGKVGDQILLAIKGQKKKALIVGHCMPGPRMTPRFDSNNVVLIEDNGNPVGTRIKTPIPTSLRKREGEYSKVLAIAQNFV>MRPL15MAGPLQGGGARALDLLRGLPRVSLANLKPNPGSKKPERRPRGRRRGRKCGRGHKGERQRGTRPRLGFEGGQTPFYIRIPKYGFNEGHSFRRQYKPLSLNRLQYLIDLGRVDPSQPIDLTQLVNGRGVTIQPLKRDYGVQLVEEGADTFTAKVNIEVQLASELAIAAIEKNGGVVTTAFYDPRSLDIVCKPVPFFLRGQPIPKRMLPPEELVPYYTDAKNRGYLADPAKFPEARLELARKYGYILPDITKDELFKMLCTRKDPRQIFFGLAPGWVVNMADKKILKPTDENLLKYYTS>MRPL16MWRLLARASAPLLRVPLSDSWALLPASAGVKTLLPVPSFEDVSIPEKPKLRFIERAPLVPKVRREPKNLSDIRGPSTEATEFTEGNFAILALGGGYLHWGHFEMMRLTINRSMDPKNMFAIWRVPAPFKPITRKSVGHRMGGGKGAIDHYVTPVKAGRLVVEMGGRCEFEEVQGFLDQVAHKLPFAAKAVSRGTLEKMRKDQEERERNNQNPWTFERIATANMLGIRKVLSPYDLTHKGKYWGKFYMPKRV>MRPL17MRLSVAAAISHGRVFRRMGLGPESRIHLLRNLLTGLVRHERIEAPWARVDEMRGYAEKLIDYGKLGDTNERAMRMADFWLTEKDLIPKLFQVLAPRYKDQTGGYTRMLQIPNRSLDRAKMAVIEYKGNCLPPLPLPRRDSHLTLLNQLLQGLRQDLRQSQEASNHSSHTAQTPGI>MRPL18MALRSRFWGLFSVCRNPGCRFAALSTSSEPAAKPEVDPVENEAVAPEFTNRNPRNLELLSVARKERGWRTVFPSREFWHRLRVIRTQHHVEALVEHQNGKVVVSASTREWAIKKHLYSTRNVVACESIGRVLAQRCLEAGINFMVYQPTPWEAASDSMKRLQSAMTEGGVVLREPQRIYE>MRPL19MGLGRSFQAARTLLPPPASIACRVHAGPVRQQSTGPSEPGAFQPPPKPVIVDKHRPVEPERRFLSPEFIPRRGRTDPLKFQIERKDMLERRKVLHIPEFYVGSILRVTTADPYASGKISQFLGICIQRSGRGLGATFILRNVIEGQGVEICFELYNPRVQEIQVVKLEKRLDDSLLYLRDALPEYSTFDVNMKPVVQEPNQKVPVNELKVKMKPKPWSKRWERPNFNIKGIRFDLCLTEQQMKEAQKWNQPWLEFDMMREYDTSKIEAAIWKEIEASKRS>MRPL20MVFLTAQLWLRNRVTDRYFRIQEVLKHARHFRGRKNRCYRLAVRTVIRAFVKCTKARYLKKKNMRTLWINRITAASQEHGLKYPALIGNLVKCQVELNRKVLADLAIYEPKTFKSLAALASRRRHEGFAAALGDGKEPEGIFSRVVQYH>MRPL21-2MAASSLTVTLGRLASACSHSILRPSGPGAASLWSASRRFNSQSTSYLPGYVPKTSLSSPPWPEVVLPDPVEETRHHAEVVKKVNEMIVTGQYGRLFAVVHFASRQWKVTSEDLILIGNELDLACGERIRLEKVLLVGADNFTLLGKPLLGKDLVRVEATVIEKTESWPRIIMRFRKRKNFKKKRIVTTPQTVLRINSIEIAPCLL>MRPL22MAAAVLGQLGALWIHNLRSRGKLALGVLPQSYIHTSASLDISRKWEKKNKIVYPPQLPGEPRRPAEIYHCRRQIKYSKDKMWYLAKLIRGMSIDQALAQLEFNDKKGAKIIKEVLLEAQDMAVRDHNVEFRSNLYIAESTSGRGQCLKRIRYHGRGRFGIMEKVYCHYFVKLVEGPPPPPEPPKTAVAHAKEYIQQLRSRTIVHTL>MRPL23MARNVVYPLYRLGGPQLRVFRTNFFIQLVRPGVAQPEDTVQFRIPMEMTRVDLRNYLEGIYNVPVAAVRTRVQHGSNKRRDHRNVRIKKPDYKVAYVQLAHGQTFTFPDLFPEKDESPEGSAADDLYSMLEEERQQRQSSDPRRGGVPSWFGL>MRPL24-1MRLSALLALASKVTLPPHYRYGMSPPGSVADKRKNPPWIRRRPVVVEPISDEDWYLFCGDTVEILEGKDAGKQGKVVQVIRQRNWVVVGGLNTHYRYIGKTMDYRGTMIPSEAPLLHRQVKLVDPMDRKPTEIEWRFTEAGERVRVSTRSGRIIPKPEFPRADGIVPETWIDGPKDTSVEDALERTYVPCLKTLQEEVMEAMGIKETRKYKKVYWY>MRPL27-1MASVVLALRTRTAVTSLLSPTPATALAVRYASKKSGGSSKNLGGKSSGRRQGIKKMEGHYVHAGNIIATQRHFRWHPGAHVGVGKNKCLYALEEGIVRYTKEVYVPHPRNTEAVDLITRLPKGAVLYKTFVHVVPAKPEGTFKLVAML>MRPL28MPLHKYPVWLWKRLQLREGICSRLPGHYLRSLEEERTPTPVHYRPHGAKFKINPKNGQRERVEDVPIPIYFPPESQRGLWGGEGWILGQIYANNDKLSKRLKKVWKPQLFEREFYSEILDKKFTVTVTMRTLDLIDEAYGLDFYILKTPKEDLCSKFGMDLKRGMLLRLARQDPQLHPEDPERRAAIYDKYKEFAIPEEEAEWVGLTLEEAIEKQRLLEEKDPVPLFKIYVAELIQQLQQQALSEPAVVQKRASGQ>MRPL30-1MAGILRLVVQWPPGRLQTVTKGVESLICTDWIRHKFTRSRIPEKVFQASPEDHEKYGGDPQNPHKLHIVTRIKSTRRRPYWEKDIIKMLGLEKAHTPQVHKNIPSVNAKLKVVKHLIRIKPLKLPQGLPAEENMSNTCLKSTGELVVQWHLKPVEQKAHES>MRPL32MALAMLVLVVSPWSAARGVLRNYWERLLRKLPQSRPGFPSPPWGPALAVQGPAMFTEPANDTSGSKENSSLLDSIFWMAAPKNRRTIEVNRCRRRNPQKLIKVKNNIDVCPECGHLKQKHVLCAYCYEKVCKETAEIRRQIGKQEGGPFKAPTIETVVLYTGETPSEQDQGKRIIERDRKRPSWFTQN>MRPL33-1MFLSAVFFAKSKSKNILVRMVSEAGTGFCFNTKRNRLREKLTLLHYDPVVKQRVLFVEKKKIRSL>MRPL34MAVLAGSLLGPTSRSAALLGGRWLQPRAWLGFPDAWGLPTPQQARGKARGNEYQPSNIKRKNKHGWVRRLSTPAGVQVILRRMLKGRKSLSH>MRPL35-1MAASAFAGAVRAASGILRPLNILASSTYRNCVKNASLISALSTGRFSHIQTPVVSSTPRLTTSERNLTCGHTSVILNRMAPVLPSVLKLPVRSLTYFSARKGKRKTVKAVIDRFLRLHCGLWVRRKAGYKKKLWKKTPARKKRLREFVFCNKTQSKLLDKMTTSFWKRRNWYVDDPYQKYHDRTNLKV>MRPL36MANLFIRKMVNPLLYLSRHTVKPRALSTFLFGSIRGAAPVAVEPGAAVRSLLSPGLLPHLLPALGFKNKTVLKKRCKDCYLVKRRGRWYVYCKTHPRHKQRQM*>MRPL37MALASGPARRALAGSGQLGLGGFGAPRRGAYEWGVRSTRKSEPPPLDRVYEIPGLEPITFAGKMHFVPWLARPIFPPWDRGYKDPRFYRSPPLHEHPLYKDQACYIFHHRCRLLEGVKQALWLTKTKLIEGLPEKVLSLVDDPRNHIENQDECVLNVISHARLWQTTEEIPKRETYCPVIVDNLIQLCKSQILKHPSLARRICVQNSTFSATWNRESLLLQVRGSGGARLSTKDPLPTIASREEIEATKNHVLETFYPISPIIDLHECNIYDVKNDTGFQEGYPYPYPHTLYLLDKANLRPHRLQPDQLRAKMILFAFGSALAQARLLYGNDAKVLEQPVVVQSVGTDGRVFHFLVFQLNTTDLDCNEGVKNLAWVDSDQLLYQHFWCLPVIKKRVVVEPVGPVGFKPETFRKFLALYLHGAA>MRPL38MPNSDIDLSNLERLEKYRSFDRYRRRAEQEAQAPHWWRTYREYFGEKTDPKEKIDIGLPPPKVSRTQQLLERKQAIQELRANVEEERAARLRTASVPLDAVRAEWERTCGPYHKQRLAEYYGLYRDLFHGATFVPRVPLHVAYAVGEDDLMPVYCGNEVTPTEAAQAPEVTYEAEEGSLWTLLLTSLDGHLLEPDAEYLHWLLTNIPGNRVAEGQVTCPYLPPFPARGSGIHRLAFLLFKQDQPIDFSEDARPSPCYQLAQRTFRTFDFYKKHQETMTPAGLSFFQCRWDDSVTYIFHQLLDMREPVFEFVRPPPYHPKQKRFPHRQPLRYLDRYRDSHEPTYGIY>MRPL39-2MEALAMGSRALRLWLVAPGGGIKWRFIATSSASQLSPTELTEMRNDLFNKEKARQLSLTPRTEKIEVKHVGKTDPGTVFVMNKNISTPYSCAMHLSEWYCRKSILALVDGQPWDMYKPLTKSCEIKFLTFKDCDPGEVNKAYWRSCAMMMGCVIERAFKDEYMVNLVRAPEVPVISGAFCYDVVLDSKLDEWMPTKENLRSFTKDAHALIYKDLPFETLEVEAKVALEIFQHSKYKVDFIEEKASQNPERIVKLHRIGDFIDVSEGPLIPRTSICFQYEVSAVHNLQPTQPSLIRRFQGVSLPVHLRAHFTIWDKLLERSRKMTPFPILLLFTTQSFFTTSPESYLLHGTVSE>MRPL40MTASVLRSISLALRPTSGLLGTWQTQLRETHQRASLLSFWELIPMRSEPLRKKKKVDPKKDQEAKERLKRKIRKLEKATQELIPIEDFITPLKFLDKARERPQVELTFEETERRALLLKKWSLYKQQERKMERDTIRAMLEAQQEALEELQLESPKLHAEAIKRDPNLFPFEKEGPHYTPPIPNYQPPEGRYNDITKVYTQVEFKR*>MRPL41MGVLAAAARCLVRGADRMSKWTSKRGPRSFRGRKGRGAKGIGFLTSGWRFVQIKEMVPEFVVPDLTGFKLKPYVSYLAPESEETPLTAAQLFSEAVAPAIEKDFKDGTFDPDNLEKYGFEPTQEGKLFQLYPRNFLR>MRPL42-1MAVAAVKWVMSKRTILKHLFPVQNGALYCVCHKSTYSPLPDDYNCNVELALTSDGRTIVCYHPSVDIPYEHTKPIPRPDPVHNNEETHDQVLKTRLEEKVEHLEEGPMIEQLSKMFFTTKHRWYPHGRYHRCRKNLNPPKDR>MRPL43-4MTARGTPSRFLASVLHNGLGRYVQQLQRLSFSVSRDGASSRGAREFVEREVIDFARRNPGVVIYVNSRPCCVPRVVAEYLNGAVREESIHCKSVEEISTLVQKLADQSGLDVIRIRKPFHTDNPSIQGQWHPFTNKPTTFRGLRPREVQDPAPAQDAAEFLGEGAGPCWYSIVALPQKHIAIAIHPPQPAGQCHQAIGHWPETVCSCTADPPARLARPNISSVIRSSLGKYPLPS>MRPL44MASGLVRLLQQGHRCLLAPVAPKLVPPVRGVKKGFRAAFRFQKELERQRLLRCPPPPVRRSEKPNWDYHAEIQAFGHRLQENFSLDLLKTAFVNSCYIKSEEAKRQQLGIEKEAVLLNLKSNQELSEQGTSFSQTCLTQFLEDEYPDMPTEGIKNLVDFLTGEEVVCHVARNLAVEQLTLSEEFPVPPAVLQQTFFAVIGALLQSSGPERTALFIRDFLITQMTGKELFEMWKIINPMGLLVEELKKRNVSAPESRLTRQSGGTTALPLYFVGLYCDKKLIAEGPGETVLVAEEEAARVALRKLYGFTENRRPWNYSKPKETLRAEKSITAS>MRPL45MAAPIPQGFSCLSRVLGWWSRQPVLVTQSAAIVPVRTKKRFTPPIYQPKFKTEKEFMQHARKAGLVIPPEKSDRSIHLACTAGIFDAYVPPEGDARISSLSKEGLIERTERMKKTMASQVSIRRIKDYDANFKIKDFPEKAKDIFIEAHLCLNNSDHDRLHTLVTEHCFPDMTWDIKYKTVRWSFVESLEPSHVVQVRCSSMMNQGNVYGQITVRMHTRQTLAIYDRFGRLMYGQEDVPKDVLEYVVFEKQLTNPYGSWRMHTKIVPPWAPPKQPILKTVMIPGPQLKPEEEYEEAQGEAQKPQLA>MRPL46MAAPVRRTLLGVAGGWRRFERLWAGSLSSRSLALAAAPSSNGSPWRLLGALCLQRPPVVSKPLTPLQEEMASLLQQIEIERSLYSDHELRALDENQRLAKKKADLHDEEDEQDILLAQDLEDMWEQKFLQFKLGARITEADEKNDRTSLNRKLDRNLVLLVREKFGDQDVWILPQAEWQPGETLRGTAERTLATLSENNMEAKFLGNAPCGHYTFKFPQAMRTESNLGAKVFFFKALLLTGDFSQAGNKGHHVWVTKDELGDYLKPKYLAQVRRFVSDL>MRPL47-2MAAAGLALLCRRVSSALKSSRSLITPQVPACTGFFLSLLPKSTPNVTSFHQYRLLHTTLSRKGLEEFFDDPKNWGQEKVKSGAAWTCQQLRNKSNEDLHKLWYVLLKERNMLLTLEQEAKRQRLPMPSPERLDKVVDSMDALDKVVQEREDALRLLQTGQERARPGAWRRDIFGRIIWHKFKQWVIPWHLNKRYNRKRFFALPYVDHFLRLEREKRARIKARKENLERKKAKILLKKFPHLAEAQKSSLV>MRPL48MSGTLEKVLCLRNNTIFKQAFSLLRFRTSGEKPIYSVGGILLSISRPYKTKPTHGIGKYKHLIKAEEPKKKKGKVEVRAINLGTDYEYGVLNIHLTAYDMTLAESYAQYVHNLCNSLSIKVEESYAMPTKTIEVLQLQDQGSKMLLDSVLTTHERVVQISGLSATFAEIFLEIIQSSLPEGVRLSVKEHTEEDFKGRFKARPELEELLAKLK>MRPL49MAATMFRATLRGWRTGVQRGCGLRLLSQTQGPPDYPRFVESVDEYQFVERLLPATRIPDPPKHEHYPTPSGWQPPRDPPPNLPYFVRRSRMHNIPVYKDITHGNRQMTVIRKVEGDIWALQKDVEDFLSPLLGKTPVTQVNEVTGTLRIKGYFDQELKAWLLEKGF>MRPL50MAARSVSGITRRVFMWTVSGTPCREFWSRFRKEKEPVVVETVEEKKEPILVCPPLRSRAYTPPEDLQSRLESYVKEVFGSSLPSNWQDISLEDSRLKFNLLAHLADDLGHVVPNSRLHQMCRVRDVLDFYNVPIQDRSKFDELSASNLPPNLKITWSY>MRPL51MAGNLLSGAGRRLWDWVPLACRSFSLGVPRLIGIRLTLPPPKVVDRWNEKRAMFGVYDNIGILGNFEKHPKELIRGPIWLRGWKGNELQRCIRKRKMVGSRMFADDLHNLNKRIRYLYKHFNRHGKFR>MRPL52-2MAALGTVLFTGVRRLHCSVAAWAGGQWRLQQGLAANPSGYGPLTELPDWSYADGRPAPPMKGQLRRKAERETFARRVVLLSQEMDAGLQAWQLRQQKLQEEQRKQENALKPKGASLKSPLPSQ>MRPL53MAAALARLGLRPVKQVRVQFCPFEKNVESTRTFLQTVSSEKVRSTNLNCSVIADVRHDGSEPCVDVLFGDGHRLIMRGAHLTALEMLTAFASHIRARDAAGSGDKPGADTGR>MRPL54MATKRLFGATRTWAGWGAWELLNPATSGRLLARDYAKKPVMKGAKSGKGAVTSEALKDPDVCTDPVQLTTYAMGVNIYKEGQDVPLKPDAEYPEWLFEMNLGPPKTLEELDPESREYWRRLRKQNIWRHNRLSKNKRL>MRPL55-3MAAVGSLLGRLRQSTVKATGPALRRLHTSSWRADSSRASLTRVHRQAYARLYPVLLVKQDGSTIHIRYREPRRMLAMPIDLDTLSPEERRARLRKREAQLQSRKEYEQELSDDLHVERYRQFWTRTKK>MRPL56-2MYRLMSAVTARAAAPGGLASSCGRRGVHQRAGLPPLGHGWVGGLGLGLGLALGVKLAGGLRGAAPAQSPAAPDPEASPLAEPPQEQSLAPWSPQTPAPPCSRCFARAIESSRDLLHRIKDEVGAPGIVVGVSVDGKEVWSEGLGYADVENRVPCKPETVMRIASISKSLTMVALAKLWEAGKLDLDIPVQHYVPEFPEKEYEGEKVSVTTRLLISHLSGIRHYEKDIKKVKEEKAYKALKMMKENVAFEQEKEGKSNEKNDFTKFKTEQENEAKCRNSKPGKKKNDFEQGELYLREKFENSIESLRLFKNDPLFFKPGSQFLYSTFGYTLLAAIVERASGCKYLDYMQKIFHDLDMLTTVQEENEPVIYNRARFYVYNKKKRLVNTPYVDNSYKWAGGGFLSTVGDLLKFGNAMLYGYQVGLFKNSNENLLPGYLKPETMVMMWTPVPNTEMSWDKEGKYAMAWGVVERKQTYGSCRKQRHYASHTGGAVGASSVLLVLPEELDTETINNKVPPRGIIVSIICNMQSVGLNSTALKIALEFDKDRSDCytosolic Ribosome>RPSAMSGALDVLQMKEEDVLKFLAAGTHLGGTNLDFQMEQYIYKRKSDGIYIINLKRTWEKLLLAARAIVAIENPADVSVISSRNTGQRAVLKFAAATGATPIAGRFTPGTFTNQIQAAFREPRLLVVTDPRADHQPLTEASYVNLPTIALCNTDSPLRYVDIAIPCNNKGAHSVGLMWWMLAREVLRMRGTISREHPWEVMPDLYFYRDPEEIEKEEQAAAEKAVTKEEFQGEWTAPAPEFTATQPEVADWSEGVQVPSVPIQQFPTEDWSAQPATEDWSAAPTAQATEWVGATTDWS>RPS2MADDAGAAGGPGGPGGPGMGNRGGFRGGFGSGIRGRGRGRGRGRGRGRGARGGKAEDKEWMPVTKLGRLVKDMKIKSLEEIYLFSLPIKESEIIDFFLGASLKDEVLKIMPVQKQTRAGQRTRFKAFVAIGDYNGHVGLGVKCSKEVATAIRGAIILAKLSIVPVRRGYWGNKIGKPHTVPCKVTGRCGSVLVRLIPAPRGTGIVSAPVPKKLLMMAGIDDCYTSARGCTATLGNFAKATFDAISKTYSYLTPDLWKETVFTKSPYQEFTDHLVKTHTRVSVQRTQAPAVATT>RPS3MAVQISKKRKFVADGIFKAELNEFLTRELAEDGYSGVEVRVTPTRTEIIILATRTQNVLGEKGRRIRELTAVVQKRFGFPEGSVELYAEKVATRGLCAIAQAESLRYKLLGGLAVRRACYGVLRFIMESGAKGCEVVVSGKLRGQRAKSMKFVDGLMIHSGDPVNYYVDTAVRHVLLRQGVLGIKVKIMLPWDPTGKIGPKKPLPDHVSIVEPKDEILPTTPISEQKGGKPEPPAMPQPVPTA>RPS3AMAVGKNKRLTKGGKKGAKKKVVDPFSKKDWYDVKAPAMFNIRNIGKTLVTRTQGTKIASDGLKGRVFEVSLADLQNDEVAFRKFKLITEDVQGKNCLTNFHGMDLTRDKMCSMVKKWQTMIEAHVDVKTTDGYLLRLFCVGFTKKRNNQIRKTSYAQHQQVRQIRKKMMEIMTREVQTNDLKEVVNKLIPDSIGKDIEKACQSIYPLHDVFVRKVKMLKKPKFELGKLMELHGEGSSSGKATGDETGAKVERADGYEPPVQESV>RPS4XMARGPKKHLKRVAAPKHWMLDKLTGVFAPRPSTGPHKLRECLPLIIFLRNRLKYALTGDEVKKICMQRFIKIDGKVRTDITYPAGFMDVISIDKTGENFRLIYDTKGRFAVHRITPEEAKYKLCKVRKIFVGTKGIPHLVTHDARTIRYPDPLIKVNDTIQIDLETGKITDFIKFDTGNLCMVTGGANLGRIGVITNRERHPGSFDVVHVKDANGNSFATRLSNIFVIGKGNKPWISLPRGKGIRLTIAEERDKRLAAKQSSG>RPS5MTEWETAAPAVAETPDIKLFGKWSTDDVQINDISLQDYIAVKEKYAKYLPHSAGRYAAKRFRKAQCPIVERLTNSMMMHGRNNGKKLMTVRIVKHAFEIIHLLTGENPLQVLVNAIINSGPREDSTRIGRAGTVRRQAVDVSPLRRVNQAIWLLCTGAREAAFRNIKTIAECLADELINAAKGSSNSYAIKKKDELERVAKSNR>RPS6MKLNISFPATGCQKLIEVDDERKLRTFYEKRMATEVAADALGEEWKGYVVRISGGNDKQGFPMKQGVLTHGRVRLLLSKGHSCYRPRRTGERKRKSVRGCIVDANLSVLNLVIVKKGEKDIPGLTDTTVPRRLGPKRASRIRKLFNLSKEDDVRQYVVRKPLNKEGKKPRTKAPKIQRLVTPRVLQHKRRRIALKKQRTKKNKEEAAEYAKLLAKRMKEAKEKRQEQIAKRRRLSSLRASTSKSESSQK>RPS7MFSSSAKIVKPNGEKPDEFESGISQALLELEMNSDLKAQLRELNITAAKEIEVGGGRKAIIIFVPVPQLKSFQKIQVRLVRELEKKFSGKHVVFIAQRRILPKPTRKSRTKNKQKRPRSRTLTAVHDAILEDLVFPSEIVGKRIRVKLDGSRLIKVHLDKAQQNNVEHKVETFSGVYKKLTGKDVNFEFPEFQL>RPS8MGISRDNWHKRRKTGGKRKPYHKKRKYELGRPAANTKIGPRRIHTVRVRGGNKKYRALRLDVGNFSWGSECCTRKTRIIDVVYNASNNELVRTKTLVKNCIVLIDSTPYRQWYESHYALPLGRKKGAKLTPEEEEILNKKRSKKIQKKYDERKKNAKISSLLEEQFQQGKLLACIASRPGQCGRADGYVLEGKELEFYLRKIKARKGK>RPS9MPVARSWVCRKTYVTPRRPFEKSRLDQELKLIGEYGLRNKREVWRVKFTLAKIRKAARELLTLDEKDPRRLFEGNALLRRLVRIGVLDEGKMKLDYILGLKIEDFLERRLQTQVFKLGLAKSIHHARVLIRQRHIRVRKQVVNIPSFIVRLDSQKHIDFSLRSPYGGGRPGRVKRKNAKKGQGGAGAGDDEEED>RPS10MLMPKKNRIAIYELLFKEGVMVAKKDVHMPKHPELADKNVPNLHVMKAMQSLKSRGYVKEQFAWRHFYWYLTNEGIQYLRDYLHLPPEIVPATLRRSRPETGRPRPKGLEGERPARLTRGEADRDTYRRSAVPPGADKKAEAGAGSATEFQFRGGFGRGRGQPPQ>RPS11MADIQTERAYQKQPTIFQNKKRVLLGETGKEKLPRYYKNIGLGFKTPKEAIEGTYIDKKCPFTGNVSIRGRILSGVVTKMKMQRTIVIRRDYLHYIRKYNRFEKRHKNMSVHLSPCFRDVQIGDIVTVGECRPLSKTVRFNVLKVTKAAGTKKQFQKF>RPS12MAEEGIAAGGVMDVNTALQEVLKTALIHDGLARGIREAAKALDKRQAHLCVLASNCDEPMYVKLVEALCAEHQINLIKVDDNKKLGEWVGLCKIDREGKPRKVVGCSCVVVKDYGKESQAKDVIEEYFKCKK>RPS13MGRMHAPGKGLSQSALPYRRSVPTWLKLTSDDVKEQIYKLAKKGLTPSQIGVILRDSHGVAQVRFVTGNKILRILKSKGLAPDLPEDLYHLIKKAVAVRKHLERNRKDKDAKFRLILIESRIHRLARYYKTKRVLPPNWKYESSTASALVA>RPS14MAPRKGKEKKEEQVISLGPQVAEGENVFGVCHIFASFNDTFVHVTDLSGKETICRVTGGMKVKADRDESSPYAAMLAAQDVAQRCKELGITALHIKLRATGGNRTKTPGPGAQSALRALARSGMKIGRIEDVTPIPSDSTRRKGGRRGRRL>RPS15MAEVEQKKKRTFRKFTYRGVDLDQLLDMSYEQLMQLYSARQRRRLNRGLRRKQHSLLKRLRKAKKEAPPMEKPEVVKTHLRDMIILPEMVGSMVGVYNGKTFNQVEIKPEMIGHYLGEFSITYKPVKHGRPGIGATHSSRFIPLK>RPS15AMVRMNVLADALKSINNAEKRGKRQVLIRPCSKVIVRFLTVMMKHGYIGEFEIIDDHRAGKIVVNLTGRLNKCGVISPRFDVQLKDLEKWQNNLLPSRQFGFIVLTTSAGIMDHEEARRKHTGGKILGFFF>RPS16MPSKGPLQSVQVFGRKKTATAVAHCKRGNGLIKVNGRPLEMIEPRTLQYKLLEPVLLLGKERFAGVDIRVRVKGGGHVAQIYAIRQSISKALVAYYQKYVDEASKKEIKDILIQYDRTLLVADPRRCESKKFGGPGARARYQKSYR*>RPS17MGRVRTKTVKKAARVIIEKYYTRLGNDFHTNKRVCEEIAIIPSKKLRNKIAGYVTHLMKRIQRGPVRGISIKLQEEERERRDNYVPEVSALDQEIIEVDPDTKEMLKLLDFGSLSNLQVTQPTVGMNFKTPRGPV>RPS18MSLVIPEKFQHILRVLNTNIDGRRKIAFAITAIKGVGRRYAHVVLRKADIDLTKRAGELTEDEVERVITIMQNPRQYKIPDWFLNRQKDVKDGKYSQVLANGLDNKLREDLERLKKIRAHRGLRHFWGLRVRGQHTKTTGRRGRTVGVSKKK>RPS19MPGVTVKDVNQQEFVRALAAFLKKSGKLKVPEWVDTVKLAKHKELAPYDENWFYTRAASTARHLYLRGGAGVGSMTKIYGGRQRNGVMPSHFSRGSKSVARRVLQALEGLKMVEKDQDGGRKLTPQGQRDLDRIAGQVAAANKKH>RPS20MAFKDTGKTPVEPEVAIHRIRITLTSRNVKSLEKVCADLIRGAKEKNLKVKGPVRMPTKTLRITTRKTPCGEGSKTWDRFQMRIHKRLIDLHSPSEIVKQITSISIEPGVEVEVTIADA>RPS21MQNDAGEFVDLYVPRKCSASNRIIGAKDHASIQMNVAEVDKVTGRFNGQFKTYAICGAIRRMGESDDSILRLAKADGIVSKNF>RPS23MGKCRGLRTARKLRSHRRDQKWHDKQYKKAHLGTALKANPFGGASHAKGIVLEKVGVEAKQPNSAIRKCVRVQLIKNGKKITAFVPNDGCLNFIEENDEVLVAGFGRKGHAVGDIPGVRFKVVKVANVSLLALYKGKKERPRS>RPS24MNDTVTIRTRKFMTNRLLQRKQMVIDVLHPGKATVPKTEIREKLAKMYKTTPDVIFVFGFRTHFGGGKTTGFGMIYDSLDYAKKNEPKHRLARHGLYEKKKTSRKQRKERKNRMKKVRGTAKANVGAGKKPKE>RPS25MPPKDDKKKKDAGKSAKKDKDPVNKSGGKAKKKKWSKGKVRDKLNNLVLFDKATYDKLCKEVPNYKLITPAVVSERLKIRGSLARAALQELLSKGLIKLVSKHRAQVIYTRNTKGGDAPAAGEDA>RPS26MTKKRRNNGRAKKGRGHVQPIRCTNCARCVPKDKAIKKFVIRNIVEAAAVRDISEASVFDAYVLPKLYVKLHYCVSCAIHSKVVRNRSREARKDRTPPPRFRPAGAAPRPPPKPM>RPS27MPLAKDLLHPSPEEEKRKHKKKRLVQSPNSYFMDVKCPGCYKITTVFSHAQTVVLCVGCSTVLCQPTGGKARLTEGCSFRRKQH>RPS27AMQIFVKTLTGKTITLEVEPSDTIENVKAKIQDKEGIPPDQQRLIFAGKQLEDGRTLSDYNIQKESTLHLVLRLRGGAKKRKKKSYTTPKKNKHKRKKVKLAVLKYYKVDENGKISRLRRECPSDECGAGVFMASHFDRHYCGKCCLTYCFNKPEDK>RPS28MDTSRVQPIKLARVTKVLGRTGSQGQCTQVRVEFMDDTSRSIIRNVKGPVREGDVLTLLESEREARRLR>RPS29MGHQQLYWSHPRKFGQGSRSCRVCSNRHGLIRKYGLNMCRQCFRQYAKDIGFIKLD>RPS30MQLFVRAQELHTFEVTGQETVAQIKAHVASLEGIAPEDQVVLLAGAPLEDEATLGQCGVEALTTLEVAGRMLGGKVHGSLARAGKVRGQTPKVAKQEKKKKKTGRAKRRMQYNRRFVNVVPTFGKKKGPNANS>RPL3MSHRKFSAPRHGSLGFLPRKRSSRHRGKVKSFPKDDPSKPVHLTAFLGYKAGMTHIVREVDRPGSKVNKKEVVEAVTIVETPPMVVVGIVGYVETPRGLRTFKTVFAEHISDECKRRFYKNWHKSKKKAFTKYCKKWQDEDGKKQLEKDFSSMKKYCQVIRVIAHTQMRLLPLRQKKAHLMEIQVNGGTVAEKLDWARERLEQQVPVNQVFGQDEMIDVIGVTKGKGYKGVTSRWHTKKLPRKTHRGLRKVACIGAWHPARVAFSVARAGQKGYHHRTEINKKIYKIGQGYLIKDGKLIKNNASTDYDLSDKSINPLGGFVHYGEVTNDFVMLKGCVVGTKKRVLTLRKSLLVQTKRRALEKIDLKFIDTTSKFGHGRFQTMEEKKAFMGPLKKDRIAKEEGA>RPL4MACARPLISVYSEKGESSGKNVTLPAVFKAPIRPDIVNFVHTNLRKNNRQPYAVSELAGHQTSAESWGTGRAVARIPRVRGGGTHRSGQGAFGNMCRGGRMFAPTKTWRRWHRRVNTTQKRYAICSALAASALPALVMSKGHRIEEVPELPLVVEDKVEGYKKTKEAVLLLKKLKAWNDIKKVYASQRMRAGKGKMRNRRRIQRRGPCIIYNEDNGIIKAFRNIPGITLLNVSKLNILKLAPGGHVGRFCIWTESAFRKLDELYGTWRKAASLKSNYNLPMHKMINTDLSRILKSPEIQRALRAPRKKIHRRVLKKNPLKNLRIMLKLNPYAKTMRRNTILRQARNHKLRVDKAAAAAAALQAKSDEKAAVAGKKPVVGKKGKKAAVGVKKQKKPLVGKKAAATKKPAPEKKPAEKKPTTEEKKPAA>RPL5MGFVKVVKNKAYFKRYQVKFRRRREGKTDYYARKRLVIQDKNKYNTPKYRMIVRVTNRDIICQIAYARIEGDMIVCAAYAHELPKYGVKVGLTNYAAAYCTGLLLARRLLNRFGMDKIYEGQVEVTGDEYNVESIDGQPGAFTCYLDAGLARTTTGNKVFGALKGAVDGGLSIPHSTKRFPGYDSESKEFNAEVHRKHIMGQNVADYMRYLMEEDEDAYKKQFSQYIKNSVTPDMMEEMYKKAHAAIRENPVYEKKPKKEVKKKRWNRPKMSLAQKKDRVAQKKASFLRAQERAAES>RPL6MAGEKVEKPDTKEKKPEAKKVDAGGKVKKGNLKAKKPKKGKPHCSRNPVLVRGIGRYSRSAMYSRKAMYKRKYSAAKSKVEKKKKEKVLATVTKPVGGDKNGGTRVVKLRKMPRYYPTEDVPRKLLSHGKKPFSQHVRKLRASITPGTILIILTGRHRGKRVVFLKQLASGLLLVTGPLVLNRVPLRRTHQKFVIATSTKIDISNVKIPKHLTDAYFKKKKLRKPRHQEGEIFDTEKEKYEITEQRKIDQKAVDSQILPKIKAIPQLQGYLRSVFALTNGIYPHKLVF>RPL7MEGVEEKKKEVPAVPETLKKKRRNFAELKIKRLRKKFAQKMLRKARRKLIYEKAKHYHKEYRQMYRTEIRMARMARKAGNFYVPAEPKLAFVIRIRGINGVSPKVRKVLQLLRLRQIFNGTFVKLNKASINMLRIVEPYIAWGYPNLKSVNELIYKRGYGKINKKRIALTDNALIARSLGKYGIICMEDLIHEIYTVGKRFKEANNFLWPFKLSSPRGGMKKKTTHFVEGGDAGNREDQINRLIRRMN>RPL7AMPKGKKAKGKKVAPAPAVVKKQEAKKVVNPLFEKRPKNFGIGQDIQPKRDLTRFVKWPRYIRLQRQRAILYKRLKVPPAINQFTQALDRQTATQLLKLAHKYRPETKQEKKQRLLARAEKKAAGKGDVPTKRPPVLRAGVNTVTTLVENKKAQLVVIAHDVDPIELVVFLPALCRKMGVPYCIIKGKARLGRLVHRKTCTTVAFTQVNSEDKGALAKLVEAIRTNYNDRYDEIRRHWGGNVLGPKSVARIAKLEKAKAKELATKLG>RPL8MGRVIRGQRKGAGSVFRAHVKHRKGAARLRAVDFAERHGYIKGIVKDIIHDPGRGAPLAKVVFRDPYRFKKRTELFIAAEGIHTGQFVYCGKKAQLNIGNVLPVGTMPEGTIVCCLEEKPGDRGKLARASGNYATVISHNPETKKTRVKLPSGSKKVISSANRAVVGVVAGGGRIDKPILKAGRAYHKYKAKRNCWPRVRGVAMNPVEHPFGGGNHQHIGKPSTIRRDAPAGRKVGLIAARRTGRLRGTKTVQEKEN>RPL9MKTILSNQTVDIPENVDITLKGRTVIVKGPRGTLRRDFNHINVELSLLGKKKKRLRVDKWWGNRKELATVRTICSHVQNMIKGVTLGFRYKMRSVYAHFPINVVIQENGSLVEIRNFLGEKYIRRVRMRPGVACSVSQAQKDELILEGNDIELVSNSAALIQQATTVKNKDIRKFLDGIYVSEKGTVQQADE>RPL10MGRRPARCYRYCKNKPYPKSRFCRGVPDAKIRIFDLGRKKAKVDEFPLCGHMVSDEYEQLSSEALEAARICANKYMVKSCGKDGFHIRVRLHPFHVIRINKMLSCAGADRLQTGMRGAFGKPQGTVARVHIGQVIMSIRTKLQNKEHVIEALRRAKFKFPGRQKIHISKKWGFTKFNADEFEDMVAEKRLIPDGCGVKYIPSRGPLDKWRALHS>RPL10AMSSKVSRDTLYEAVREVLHGNQRKRRKFLETVELQISLKNYDPQKDKRFSGTVRLKSTPRPKFSVCVLGDQQHCDEAKAVDIPHMDIEALKKLNKNKKLVKKLAKKYDAFLASESLIKQIPRILGPGLNKAGKFPSLLTHNENMVAKVDEVKSTIKFQMKKVLCLAVAVGHVKMTDDELVYNIHLAVNFLVSLLKKNWQNVRALYIKSTMGKPQRLY>RPL11MAQDQGEKENPMRELRIRKLCLNICVGESGDRLTRAAKVLEQLTGQTPVFSKARYTVRSFGIRRNEKIAVHCTVRGAKAEEILEKGLKVREYELRKNNFSDTGNFGFGIQEHIDLGIKYDPSIGIYGLDFYVVLGRPGFSIADKKRRTGCIGAKHRISKEEAMRWFQQKYDGIILPGK>RPL12MPPKFDPNEIKVVYLRCTGGEVGATSALAPKIGPLGLSPKKVGDDIAKATGDWKGLRITVKLTIQNRQAQIEVVPSASALIIKALKEPPRDRKKQKNIKHSGNITFDEIVNIARQMRHRSLARELSGTIKEILGTAQSVGCNVDGRHPHDIIDDINSGAVECPAS>RPL13MAPSRNGMVLKPHFHKDWQRRVATWFNQPARKIRRRKARQAKARRIAPRPASGPIRPIVRCPTVRYHTKVRAGRGFSLEELRVAGIHKKVARTIGISVDPRRRNKSTESLQANVQRLKEYRSKLILFPRKPSAPKKGDSSAEELKLATQLTGPVMPVRNVYKKEKARVITEEEKNFKAFASLRMARANARLFGIRAKRAKEAAEQDVEKKK>RPL13AMAEVQVLVLDGRGHLLGRLAAIVAKQVLLGRKVVVVRCEGINISGNFYRNKLKYLAFLRKRMNTNPSRGPYHFRAPSRIFWRTVRGMLPHKTKRGQAALDRLKVFDGIPPPYDKKKRMVVPAALKVVRLKPTRKFAYLGRLAHEVGWKYQAVTATLEEKRKEKAKIHYRKKKQLMRLRKQAEKNVEKKIDKYTEVLKTHGLLV>RPL14MVFRRFVEVGRVAYVSFGPHAGKLVAIVDVIDQNRALVDGPCTQVRRQAMPFKCMQLTDFILKFPHSAHQKYVRQAWQKADINTKWAATRWAKKIEARERKAKMTDFDRFKVMKAKKMRNRIIKNEVKKLQKAALLKASPKKAPGTKGTAAAAAAAAAAKVPAKKITAASKKAPAQKVPAQKATGQKAAPAPKAQKGQKAPAQKAPAPKASGKKA>RPL15MGAYKYIQELWRKKQSDVMRFLLRVRCWQYRQLSALHRAPRPTRPDKARRLGYKAKQGYVIYRIRVRRGGRKRPVPKGATYGKPVHHGVNQLKFARSLQSVAEERAGRHCGALRVLNSYWVGEDSTYKFFEVILIDPFHKAIRRNPDTQWITKPVHKHREMRGLTSAGRKSRGLGKGHKFHHTIGGSRRAAWRRRNTLQLHRYR>RPL17MVRYSLDPENPTKSCKSRGSNLRVHFKNTRETAQAIKGMHIRKATKYLKDVTLQKQCVPFRRYNGGVGRCAQAKQWGWTQGRWPKKSAEFLLHMLKNAESNAELKGLDVDSLVIEHIQVNKAPKMRRRTYRAHGRINPYMSSPCHIEMILTEKEQIVPKPEEEVAQKKKISQKKLKKQKLMARE>RPL18MGVDIRHNKDRKVRRKEPKSQDIYLRLLVKLYRFLARRTNSTFNQVVLKRLFMSRTNRPPLSLSRMIRKMKLPGRENKTAVVVGTITDDVRVQEVPKLKVCALRVTSRARSRILRAGGKILTFDQLALDSPKGCGTVLLSGPRKGREVYRHFGKAPGTPHSHTKPYVRSKGRKFERARGRRASRGYKN>RPL18AMKASGTLREYKVVGRCLPTPKCHTPPLYRMRIFAPNHVVAKSRFWYFVSQLKKMKKSSGEIVYCGQVFEKSPLRVKNFGIWLRYDSRSGTHNMYREYRDLTTAGAVTQCYRDMGARHRARAHSIQIMKVEEIAASKCRRPAVKQFHDSKIKFPLPHRVLRRQHKPRFTTKRPNTFF>RPL19MSMLRLQKRLASSVLRCGKKKVWLDPNETNEIANANSRQQIRKLIKDGLIIRKPVTVHSRARCRKNTLARRKGRHMGIGKRKGTANARMPEKVTWMRRMRILRRLLRRYRESKKIDRHMYHSLYLKVKGNVFKNKRILMEHIHKLKADKARKKLLADQAEARRSKTKEARKRREERLQAKKEEIIKTLSKEEETKK>RPL21MTNTKGKRRGTRYMFSRPFRKHGVVPLATYMRIYKKGDIVDIKGMGTVQKGMPHKCYHGKTGRVYNVTQHAVGIVVNKQVKGKILAKRINVRIEHIKHSKSRDSFLKRVKENDQKKKEAKEKGTWVQLKRQPAPPREAHFVRTNGKEPELLEPIPYEFMA>RPL22MAPVKKLVVKGGKKKKQVLKFTLDCTHPVEDGIMDAANFEQFLQERIKVNGKAGNLGGGVVTIERSKSKITVTSEVPFSKRYLKYLTKKYLKKNNLRDWLRVVANSKESYELRYFQINQDEEEEEDED>RPL23MSKRGRGGSSGAKFRISLGLPVGAVINCADNTGAKNLYIISVKGIKGRLNRLPAAGVGDMVMATVKKGKPELRKKVHPAVVIRQRKSYRRKDGVFLYFEDNAGVIVNNKGEMKGSAITGPVAKECADLWPRIASNAGSIA>RPL23AMAPKAKKEAPAPPKAEAKAKALKAKKAVLKGVHSHKKKKIRTSPTFRRPKTLRLRRQPKYPRKSAPRRNKLDHYAIIKFPLTTESAMKKIEDNNTLVFIVDVKANKHQIKQAVKKLYDIDVAKVNTLIRPDGEKKAYVRLAPDYDALDVANKIGII>RPL24MKVELCSFSGYKIYPGHGRRYARTDGKVFQFLNAKCESAFLSKRNPRQINWTVLYRRKHKKGQSEEIQKKRTRRAVKFQRAITGASLADIMAKRNQKPEVRKAQREQAIRAAKEAKKAKQASKKTAMAAAKAPTKAAPKQKIVKPVKVSAPRVGGKR>RPL26MKFNPFVTSDRSKNRKRHFNAPSHIRRKIMSSPLSKELRQKYNVRSMPIRKDDEVQVVRGHYKGQQIGKVVQVYRKKYVIYIERVQREKANGTTVHVGIHPSKVVITRLKLDKDRKKILERKAKSRQVGKEKGKYKEETIEKMQE>RPL27MGKFMKPGKVVLVLAGRYSGRKAVIVKNIDDGTSDRPYSHALVAGIDRYPRKVTAAMGKKKIAKRSKIKSFVKVYNYNHLMPTRYSVDIPLDKTVVNKDVFRDPALKRKARREAKVKFEERYKTGKNKWFFQKLRF>RPL27AMPSRLRKTRKLRGHVSHGHGRIGKHRKHPGGRGNAGGLHHHRINFDKYHPGYFGKVGMKHYHLKRNQSFCPTVNLDKLWTLVSEQTRVNAAKNKTGAAPIIDVVRSGYYKVLGKGKLPKQPVIVKAKFFSRRAEEKIKSVGGACVLVA>RPL28MSAHLQWMVVRNCSSFLIKRNKQTYSTEPNNLKARNSFRYNGLIHRKTVGVEPAADGKGVVVVIKRRSGQRKPATSYVRTTINKNARATLSSIRHMIRKNKYRPDLRMAAIRRASAILRSQKPVMVKRKRTRPTKSS>RPL29MAKSKNHTTHNQSRKWHRNGIKKPRSQRYESLKGVDPKFLRNMRFAKKHNKKGLKKMQANNAKAMSARAEAIKALVKPKEVKPKIPKGVSRKLDRLAYIAHPKLGKRARARIAKGLRLCRPKAKAKAKDQTKAQAAAPASVPAQAPKRTQAPTKASE>RPL30MVAAKKTKKSLESINSRLQLVMKSGKYVLGYKQTLKMIRQGKAKLVILANNCPALRKSEIEYYAMLAKTGVHHYSGNNIELGTACGKYYRVCTLAIIDPGDSDIIRSMPEQTGEK>RPL31MAPAKKGGEKKKGRSAINEVVTREYTINIHKRIHGVGFKKRAPRALKEIRKFAMKEMGTPDVRIDTRLNKAVWAKGIRNVPYRIRVRLSRKRNEDEDSPNKLYTLVTYVPVTTFKNLQTVNVDEN>RPL32MAALRPLVKPKIVKKRTKKFIRHQSDRYVKIKRNWRKPRGIDNRVRRRFKGQILMPNIGYGSNKKTKHMLPSGFRKFLVHNVKELEVLLMCNKSYCAEIAHNVSSKNRKAIVERAAQLAIRVTNPNARLRSEENE>RPL34MVQRLTYRRRLSYNTASNKTRLSRTPGNRIVYLYTKKVGKAPKSACGVCPGRLRGVRAVRPKVLMRLSKTKKHVSRAYGGSMCAKCVRDRIKRAFLIEEQKIVVKVLKAQAQSQKAK>RPL35MAKIKARDLRGKKKEELLKQLDDLKVELSQLRVAKVTGGAASKLSKIRVVRKSIARVLTVINQTQKENLRKFYKGKKYKPLDLRPKKTRAMRRRLNKHEENLKTKKQQRKERLYPLRKYAVKA>RPL35AMSGRLWSKAIFAGYKRGLRNQREHTALLKIEGVYARDETEFYLGKRCAYVYKAKNNTVTPGGKPNKTRVIWGKVTRAHGNSGMVRAKFRSNLPAKAIGHRIRVMLYPSRI>RPL36MALRYPMAVGLNKGHKVTKNVSKPRHSRRRGRLTKHTKFVRDMIREVCGFAPYERRAMELLKVSKDKRALKFIKKRVGTHIRAKRKREELSNVLAAMRKAAAKKD>RPL36AMVNVPKTRRTFCKKCGKHQPHKVTQYKKGKDSLYAQGKRRYDRKQSGYGGQTKPIFRKKAKTTKKIVLRLECVEPNCRSKRMLAIKRCKHFELGGDKKRKGQVIQF>RPL37MTKGTSSFGKRRNKTHTLCRRCGSKAYHLQKSTCGKCGYPAKRKRKYNWSAKAKRRNTTGTGRMRHLKIVYRRFRHGFREGTTPKPKRAAVAASSSS>RPL37AMAKRTKKVGIVGKYGTRYGASLRKMVKKIEISQHAKYTCSFCGKTKMKRRAVGIWHCGSCMKTVAGGAWTYNTTSAVTVKSAIRRLKELKDQ>RPL38MPRKIEEIKDFLLTARRKDAKSVKIKKNKDNVKFKVRCSRYLYTLVITDKEKAEKLKQSLPPGLAVKELK>RPL39MSSHKTFRIKRFLAKKQKQNRPIPQWIRMKTGNKIRYNSKRRHWRRTKLGL>RPL40MQIFVKTLTGKTITLEVEPSDTIENVKAKIQDKEGIPPDQQRLIFAGKQLEDGRTLSDYNIQKESTLHLVLRLRGGIIEPSLRQLAQKYNCDKMICRKCYARLHPRAVNCRKKKCGHTNNLRPKKKVK>RPL41MRAKWRKKRMRRLKRKRRKMRQRSK
