# Supplementary material for: P53-dependent hypusination of eIF5A affects mitochondrial translation and senescence immune surveillance
Source: Nat Commun. 2024 Aug 28;15:7458. doi: 10.1038/s41467-024-51901-w (PMC11358140; doi:10.1038/s41467-024-51901-w)
Supplement: Supplementary file 9 — Reporting Summary [file 41467_2024_51901_MOESM9_ESM.pdf]

nature portfolio

Corresponding author(s): Fabricio Loayza-Puch

Last updated by author(s): Apr 5, 2024

Reporting Summary

Nature Portfolio wishes to improve the reproducibility of the work that we publish. This form provides structure for consistency and transparency in reporting. For further information on Nature Portfolio policies, see our Editorial Policies and the Editorial Policy Checklist.

Statistics

For all statistical analyses, confirm that the following items are present in the figure legend, table legend, main text, or Methods section.

n/a	Confirmed	
☐	☒	The exact sample size (*n*) for each experimental group/condition, given as a discrete number and unit of measurement
☒	☐	A statement on whether measurements were taken from distinct samples or whether the same sample was measured repeatedly
☐	☒	The statistical test(s) used AND whether they are one- or two-sided<br>*Only common tests should be described solely by name; describe more complex techniques in the Methods section.*
☐	☒	A description of all covariates tested
☒	☐	A description of any assumptions or corrections, such as tests of normality and adjustment for multiple comparisons
☐	☒	A full description of the statistical parameters including central tendency (e.g. means) or other basic estimates (e.g. regression coefficient) AND variation (e.g. standard deviation) or associated estimates of uncertainty (e.g. confidence intervals)
☐	☒	For null hypothesis testing, the test statistic (e.g. *F*, *t*, *r*) with confidence intervals, effect sizes, degrees of freedom and *P* value noted<br>*Give P values as exact values whenever suitable.*
☒	☐	For Bayesian analysis, information on the choice of priors and Markov chain Monte Carlo settings
☒	☐	For hierarchical and complex designs, identification of the appropriate level for tests and full reporting of outcomes
☒	☐	Estimates of effect sizes (e.g. Cohen's *d*, Pearson's *r*), indicating how they were calculated

Our web collection on statistics for biologists contains articles on many of the points above.

Software and code

Policy information about availability of computer code

Data collection

Flow cytometric analysis was performed on a FACSCanto Analyzer (BD Biosciences, New Jersey, USA).

Sequencing libraries were prepared as previously described (see Methods section) and sequenced single-end, 100 bp, on one lane of a Nextseq 2000 sequencers (llumina).

Proteomic measurements were performed in the The QExactive HF mass spectrometer

LC-MS based metabolomics was performed on an Q-Exactive HF mass spectrometer (Thermo Scientific) coupled to a Vanquish autosampler and pump (Thermo Scientific).

qPCR data were acquired using QuantStudio 3 Real-Time PCR System.

Data analysis

Differential gene expression analysis was performed with the R package DESeq2 v1.26.0

Flow cytometry data were analyzed using FACSDiva software, Cytek Spectroflaw and Flowlo 10.4 (BD Biosciences).

Raw proteomic files were processed using Maxquant version 2.0.1 and 2.0.3 and the Andromeda search engine. Details on the proteomic data analysis are provided in the manuscript.

LC-MS based metabolomics quantification was based on peak area using TraceFinder software (Thermo Scientific)

For manuscripts utilizing custom algorithms or software that are central to the research but not yet described in published literature, software must be made available to editors and reviewers. We strongly encourage code deposition in a community repository (e.g. GitHub). See the Nature Portfolio guidelines for submitting code & software for further information.

Data

Policy information about availability of data

All manuscripts must include a data availability statement. This statement should provide the following information, where applicable:

- Accession codes, unique identifiers, or web links for publicly available datasets
- A description of any restrictions on data availability
- For clinical datasets or third party data, please ensure that the statement adheres to our policy

A complete data availability statement is included in the manuscript

Research involving human participants, their data, or biological material

Policy information about studies with human participants or human data. See also policy information about sex, gender (identity/presentation), and sexual orientation and race, ethnicity and racism.

Reporting on sex and gender	n/a
Reporting on race, ethnicity, or other socially relevant groupings	n/a
Population characteristics	n/a
Recruitment	n/a
Ethics oversight	n/a

Note that full information on the approval of the study protocol must also be provided in the manuscript.

Field-specific reporting

Please select the one below that is the best fit for your research. If you are not sure, read the appropriate sections before making your selection.

☒ Life sciences ☐ Behavioural & social sciences ☐ Ecological, evolutionary & environmental sciences

For a reference copy of the document with all sections, see nature.com/documents/nr-reporting-summary-flat.pdf

Life sciences study design

All studies must disclose on these points even when the disclosure is negative.

Sample size	Sample sizes were determined based on prior knowledge of good sample sizes to ensure adequate data for reliable assessments. Sample sizes are indicated in the figure legends.
Data exclusions	Data was excluded based on QC results for individual assays.
Replication	All experiments were performed in at least three biological replicates and specific sample sizes are mentioned in the figure legends. Most experiments contain statistical analysis and significances of the results are indicated.
Randomization	Animal experiments were performed in randomized order.
Blinding	The investigators were not blinded to group allocation due to fixed experimental groups used for comparison.

Reporting for specific materials, systems and methods

We require information from authors about some types of materials, experimental systems and methods used in many studies. Here, indicate whether each material, system or method listed is relevant to your study. If you are not sure if a list item applies to your research, read the appropriate section before selecting a response.

Materials & experimental systems

n/a	Involved in the study
	☒ Antibodies
	☒ Eukaryotic cell lines
☒	Palaeontology and archaeology
	☒ Animals and other organisms
☒	Clinical data
☒	Dual use research of concern
☒	Plants

Methods

n/a	Involved in the study
☒	ChIP-seq
	☒ Flow cytometry
☒	MRI-based neuroimaging

Antibodies

Antibodies used	eIF5A antibody was from BD Transduction Laboratories (611977; 1:10,000; https://www.bdbiosciences.com/en-us/products/reagents/microscopy-imaging-reagents/immunofluorescence-reagents/purified-mouse-anti-eif-5a.611977).<br>Anti-hypusine eIF5A was from EMD Millipore (ABS1064-I; 1:2,000; https://www.emdmillipore.com/US/en/product/Anti-Hypusine,MM_NF-ABS1064-I-100UL)<br>Anti-MRPL11 (2199; 1:500; https://www.cellsignal.com/products/primary-antibodies/mrpl11-antibody/2199), anti-GFP (2956; 1:100; https://www.cellsignal.com/products/primary-antibodies/gfp-d5-1-rabbit-mab/2956), and anti-RPS6 (2217; 1:1,000; https://www.cellsignal.com/products/primary-antibodies/s6-ribosomal-protein-5g10-rabbit-mab/2217) were purchased from Cell Signaling Technologies.<br>Anti-RPL10a was from Abcam (ab226381; 1:1000; https://www.abcam.com/en-us/products/primary-antibodies/rpl10a-antibody-ab226381).<br>Anti-MRPL37 (15190-1-AP; 1:500; https://www.ptglab.com/products/MRPL37-Antibody-15190-1-AP.htm), anti MRPS30 (1844-1-AP; 1:500; https://www.ptglab.com/products/MRPS30-Antibody-18441-1-AP.htm), anti-GAPDH (60004; 1:20,000; https://www.ptglab.com/products/GAPDH-Antibody-HRP-60004.htm), and anti-SMOX (15052-1-AP; 1:500; https://www.ptglab.com/products/SMOX-Antibody-15052-1-AP.htm) were purchased from Proteintech.<br>Anti-CD45 was from Dako (52369; 1:100)
Validation	Antibodies were purchased from the above stated companies. Antibodies are well described and published elsewhere. information can be sought from the manufactures website under the respective catalog number.

Eukaryotic cell lines

Policy information about cell lines and Sex and Gender in Research

Cell line source(s)	BJ (ATCC ,CRL-2522), IMR90 (ATCC, CCL-186), TIG3 (CVCL_E939), A549 (ATCC, CCL-185), HeLa (ATCC, CCL-2), MCF7 (HTB-22), HCT116 (CCL-247), U2OS (HTB-96)
Authentication	All cell lines were authenticated using the Multiplex cell line authentication test by Multiplexion (Heidelberg Germany)
Mycoplasma contamination	All cell lines were controlled for contamination using the LookOut Mycoplasma PCR Detection Kit (Sigma)
Commonly misidentified lines<br>(See ICLAC register)	No commonly misidentified cell lines were used.

Animals and other research organisms

Policy information about studies involving animals; ARRIVE guidelines recommended for reporting animal research, and Sex and Gender in Research

Laboratory animals	Male C57BL/6J mice (Mus musculus), 8 weeks old were purchased from Charles River or bred in house at the DKFZ Center for Preclinical Research facility
Wild animals	No wild animal we used in this study
Reporting on sex	Because this study focuses on female breast cancer, all animals used were female.
Field-collected samples	No field-collected samples were used in this study
Ethics oversight	All mice animal studies were approved by the controlling government office (Regierungspräsidium Karlsruhe) according to the German Animal Protection Law, and in compliance with the EU Directive on animal welfare, Directive 2010/63/EU.

Note that full information on the approval of the study protocol must also be provided in the manuscript.

Plants

Seed stocks: n/a

Novel plant genotypes: n/a

Authentication: n/a

Flow Cytometry

Plots

Confirm that:

☒ The axis labels state the marker and fluorochrome used (e.g. CD4-FITC).

☒ The axis scales are clearly visible. Include numbers along axes only for bottom left plot of group (a 'group' is an analysis of identical markers).

☒ All plots are contour plots with outliers or pseudocolor plots.

☒ A numerical value for number of cells or percentage (with statistics) is provided.

Methodology

Sample preparation: Sample preparation of the individual flow cytometry experiments are described in detail in the methods part of the manuscript.

Instrument: FACS Canto II cytometer, Fortessa HTS, BD Aria II (BD Biosciences)

Software: FlowJo 10.0.8 (BD)

Cell population abundance: For the OP-puro based CRISPR screen, 10 million cells were sorted. The low OP-Puro fraction represented the 15th percentile of the OP-puro positive cells. The purity of this populations was approx. 90%

Gating strategy: For protein synthesis rate determination by op-puromycin: FSC/SSC is for population selection and doublet exclusion. A negative control (-OPP) is used to normalize the median of Alexa Fluor 488 or Alexa Fluor 647 signal intensity on the respective channels (BL-530/RL-670).

☐ Tick this box to confirm that a figure exemplifying the gating strategy is provided in the Supplementary Information.
